# Calculation Method for Hydrogen Evolution and Adsorption on Ir, Pt, and Ir–Pt Nanocatalysts Supported on Porous Carbon Electrodes in Water Electrolysis

**DOI:** 10.1021/acsomega.6c03700

**Published:** 2026-05-08

**Authors:** Farid Taherkhani, Fabian Mauss

**Affiliations:** † Departments of Thermodynamics and Thermal Process Engineering, Brandenburg University of Technology, Cottbus 03046, Germany; ‡ Energiespeicher-und Energiewandlersysteme, Brandenburgische Technische Universität Cottbus−Senftenberg, Cottbus 03046, Germany

## Abstract

The Tafel exchange current density for the electrochemical hydrogen evolution reaction (HER) in water electrolysis was systematically evaluated on iridium (Ir) nanoelectrodes using first-principles computational methods. For the first time, the transition from crystalline (metallic) to amorphous structural regimes in Ir nanoclusters is explicitly correlated with variations in exchange current density, as determined from density functional theory (DFT) calculations. The results reveal a pronounced maximum in the exchange current density associated with the structural transformation from ordered crystalline to disordered amorphous phases under acidic conditions. This enhancement is attributed to modifications in the local electronic structure and active site distribution induced by amorphization. The theoretically predicted exchange current densities are in quantitative agreement with available experimental measurements, thereby validating the computational approach. DFT calculation predicts that for small iridium nanoparticles (NPs) (20–50 atoms), the exchange current density for the HER in acidic media is around 1.1 (mA/cm^2^), and it approaches 5 mA/cm^2^ upon undergoing amorphous phase transition. The current density has also been calculated for pure Ir, pure Pt, and Ir–Pt nanoalloy electrodes designed for the HER in alkaline media. Furthermore, molecular dynamics (MD) simulation shows that Pt doping in Ir nanoclusters, supported on single-walled carbon nanotubes (SWCNTs), enhances hydrogen adsorption and increases the current density for the HER in alkaline water electrolysis, even at low Pt concentrations. The calculated current density based on DFT computation values is consistent with the published experimental data. DFT calculation estimates that the current density for the HER in alkaline media at 50 mV is 5.5, 6, and 7 (mA/cm^2^) for Ir, Pt, and the Ir–Pt nanoalloy with a total atom number of 30, respectively. Furthermore, the heat capacity (*C*_p_) of Ir@SWCNT exhibits a transition from monotonic to nonmonotonic temperature dependence with increasing Ir cluster size. MD simulations further demonstrate that the hydrogen diffusion coefficient on Ir and Pt nanoparticles (NPs) supported on single-walled carbon nanotubes (Ir@SWCNT and Pt@SWCNT) decreases with decreasing cluster size. In addition, computational analysis reveals the formation of multilayer hydrogen adsorption on both monometallic Ir and bimetallic Ir–Pt nanocatalysts supported on SWCNTs (IrPt@SWCNT), suggesting strong adsorbate–adsorbate interactions under the studied conditions.

## Graphical abstract

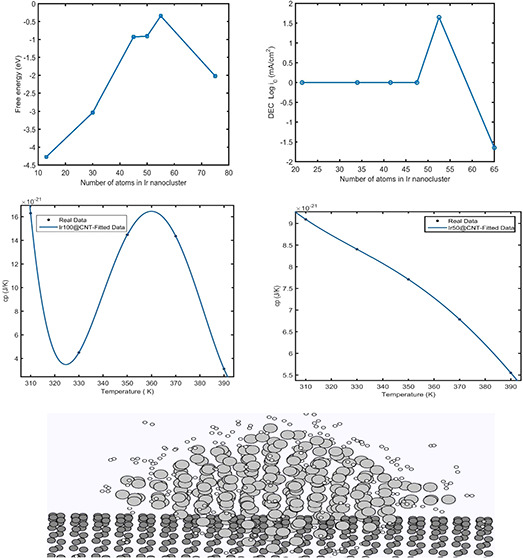

## 1 Introduction

The kinetics and mechanisms of hydrogen evolution on TiNi alloy electrodes have been investigated using steady-state potentiostatic polarization and AC impedance spectroscopy.^1^ Hydrogen evolution has also been studied on nitrogen-doped graphite containing well-dispersed Janus Co/CoP nanocrystals as active sites.^2^ Significant efforts to enhance the catalytic activity of the hydrogen evolution reaction (HER) have focused on transition metal dichalcogenides, which represent promising nonprecious metal catalysts.^3^ The HER has also been examined on platinum supported on nitrogen-doped graphite.^4^ NPs used as catalysts play a crucial role in hydrogen evolution and contribute substantially to advancing hydrogen as a sustainable alternative to fossil fuels.^5^

The HER is a key electrochemical process in which protons from solution combine with electrons at the electrode surface to form hydrogen gas.^6^ Numerous experimental studies have evaluated Ir–Pt nanoalloys for both hydrogen and oxygen evolution in water electrolysis systems,^7^ and Ir–Pt nanocatalysts have also been employed for methanol oxidation reactions.^8^ Additionally, scandium (Sc) doping in Ir–Pt nanoalloys has been shown to enhance current density during the HER in water electrolysis.^9^ Hydrogen production via water electrolysis has been demonstrated using a wide range of electrocatalysts, including Pt, Ag, Au, and Ru.^10^ Electrocatalytic Ni–Mo oxides have further exhibited HER activity in acidic, alkaline, and neutral electrolytes.^11^ Collectively, previous research highlights the central importance of electrocatalysts in improving hydrogen generation during water electrolysis,^12^ with electrode materials, electrolyte additives, and gas bubble behavior identified as key factors for further technological progress.^13^

Platinum (Pt) alloys are widely recognized for their excellent catalytic activity toward the HER. Prior studies have demonstrated that doping Pt with rare earth metals such as samarium (Sm), cerium (Ce), and holmium (Ho) substantially enhances HER performance in alkaline media.^14^ Similarly, introducing small amounts of Sm into nickel (Ni) significantly improves catalytic activity for the alkaline HER.^15^ Nickel hydroxide (Ni­(OH)_2_) has also shown promising HER activity under alkaline conditions.^16^ Density functional theory (DFT) has been extensively applied to elucidate HER mechanisms in water electrolysis,^17^ with DFT calculations used to determine hydrogen binding energies on materials such as molybdenum, nickel, silver, titanium dioxide, and platinum to assess their catalytic potential.^18^ Theoretical studies have also examined catalytic behavior in alkaline environments.^19^ Many efforts have been made to increase the current density of water electrolysis for the production of green hydrogen by using catalysis development. The creation of next-generation water electrolysis systems ensures a sustainable and profitable hydrogen economy, while also directly advancing the Sustainable Development Goals (SDGs) of the UN, which include clean energy, industrial innovation, and climate action.^20^

Previous studies indicate that Ir is highly active toward the oxygen evolution reaction (OER), whereas Pt exhibits comparatively weaker OER activity.^21^ Hydrogen adsorption has been explored using Al–Ag NPs supported on carbon nanotubes with varying defect densities, where the defect structure plays a critical role.^22^ It has also been reported that Ir NPs can undergo a transition toward an amorphous-like structure under specific conditions.^23^ Moreover, electrode fabrication using metallic NPs has been found to strongly influence current density during electrochemical reactions.^24^ Ion exchange membranes for water electrolysis have been investigated in low-concentration alkaline electrolytes.^25^

The hydrogen evolution reaction (HER) is a fundamental electrochemical process responsible for generating hydrogen gas during water splitting and plays a central role in sustainable energy technologies. Its key features include thermodynamics (with a standard potential of 0 V vs RHE), reaction kinetics (proceeding via the Volmer–Heyrovsky or Volmer–Tafel mechanisms), and catalytic activity, which is strongly governed by the hydrogen adsorption free energy (Δ*G*_H*). Efficient HER catalysts require an optimal hydrogen binding strength, high electrical conductivity, abundant active sites, and long-term operational stability. From a materials science perspective, factors such as the electronic structure, surface morphology, defect engineering, and interface design critically influence catalytic performance. A comprehensive understanding and optimization of these parameters are essential for developing cost-effective, high-performance catalysts for green hydrogen production.^26–29^

Nonprecious metal catalysts provide significant advantages in terms of cost and scalability for hydrogen production via water electrolysis. Their earth abundance and lower material costs make them particularly attractive for large-scale and industrial applications, where catalyst loading and long-term operational expenses are key considerations. However, catalysts based on iridium (Ir) and platinum (Pt) generally exhibit superior intrinsic activity, faster reaction kinetics, especially in acidic media, and enhanced electrochemical stability under harsh conditions. As a result, although nonprecious metal systems offer clear economic benefits, Ir- and Pt-based materials remain the benchmark for performance and durability, underscoring the fundamental trade-off between cost effectiveness and catalytic efficiency.

Water electrolysis can operate in both acidic and alkaline environments. One of the open research questions addressed in this study concerns the variation of current density during the HER in acidic media in water electrolysis for fabricated iridium (Ir) NPs, particularly focusing on NP size and phase transitions from crystalline to amorphous structures. Enhancing current density in alkaline media through the design of Ir-based nanoalloy electrodes remains a significant challenge. Therefore, the present research investigates the dependence of current density on overpotential for Ir, Pt, and Ir–Pt nanocatalysts fabricated as electrodes. An experimental study regarding the HER in alkaline media for water electrolysis on nanocomposite Pt, Ir, and Pt–Ir shows that the potential corresponding to the current density of 10 mA/cm^2^ exhibits an enhancement of 80 mV for a 1 min Ir/Pt­(poly) electrode with respect to Pt­(poly), which drops down to around 50 and 20 mV for Ir/Pt­(poly) electrodes obtained after 3 and 30 min of deposition, respectively.^7^

Infrared molecular spectroscopy of the Tafel step in the hydrogen evolution reaction (HER) during water electrolysis on ultrasmall iridium nanoparticles (Ir NPs) remains largely unexplored and is an active area of current research. The hydrogen adsorption behavior on Ir@SWCNT and Pt@SWCNT systems, particularly as a function of the number of atoms in Ir and Pt nanoclusters, is still an open question. Furthermore, the effects of temperature and pressure on hydrogen adsorption in Ir@SWCNT and Pt@SWCNT materials are not yet fully understood. The heat capacity as a function of temperature for varying numbers of Ir atoms in Ir@SWCNT also remains to be clarified. In addition, hydrogen diffusion behavior across nanoclusters of different sizes and compositions, specifically Ir and Pt clusters supported on SWCNTs, continues to be an important unresolved issue. Another significant research challenge is elucidating hydrogen adsorption and release mechanisms in Ir–Pt nanoalloys with varying compositions (Ir_1–*x*_Pt*_x_* @SWCNT). Understanding these processes is particularly relevant for nanoporous carbon electrodes incorporating Ir–Pt nanoalloys, where adsorption–desorption dynamics directly influence hydrogen evolution performance during water electrolysis.

## 2 Computational Methods

### 2.1 HER for Water Electrolysis in Alkaline Media

The mechanism of the HER in alkaline solutions^1^ involves the proton discharge electrosorption (Volmer reaction, reaction 1), electrochemical desorption (Heyrovsky reaction, reaction 2), and H recombination (Tafel reaction, reaction 3)

1 $$M+H_{2}O+e⇌\mathrm{MH(ad)}+OH^{-}$$

2 $$\mathrm{MH}\left(\mathrm{ad}\right)+H_{2}O+e⇌H_{2}+M+OH^{-}$$

3 $$\mathrm{MH}\left(\mathrm{ads}\right)+\mathrm{MH}\left(\mathrm{ads}\right)⇌H_{2}+2M$$

We assume that the reaction rate constants for reactions 1–3 are shown with *k*_1_, *k*_–1_, *k*_2_, *k*_–2_, *k*_3_, and *k*_–3_, respectively.

The electrochemical rate equation for the above reaction 1 is

4 $$r_{1}=k_{1}\left(1-\theta \right)\exp\left(-\beta \frac{F\eta }{\mathrm{RT}}\right)-k_{-1}\theta \;\exp\left(\frac{\left(1-\beta \right)F\eta }{\mathrm{RT}}\right)$$

The rate equation for electrochemical reaction 2 is

5 $$r_{2}=k_{2}\theta \;\exp\left(-\beta \frac{F\eta }{\mathrm{RT}}\right)-k_{-2}\left(1-\theta \right)\exp\left(\frac{\left(1-\beta \right)F\eta }{\mathrm{RT}}\right)$$

Also, the rate equation for chemical reaction 3 is

6 $$r_{3}=k_{3}\theta^{2}-k_{-3}{\left(1-\theta \right)}^{2}$$

Calculation of the current density could be performed as the following equation

7 $$j=-F\left(r_{1}+r_{2}\right)$$

8 $$\frac{q}{F}\left(\frac{d\theta }{\mathrm{dt}}\right)=\left(r_{1}-r_{2}-2r_{3}\right)$$

where *q*, *F, θ, t, r*_1_, *r*_2_, *r*_3_, *η, R*, and *T* are the charge on the surface electrode, faradic constant, time, chemical rate of reactions 1, 2, and 3 based on formation of the M-H compound, electrical overpotential, universal gas constant, and temperature, respectively.

### 2.2 HER for Water Electrolysis in Acidic Media

The electrochemical HER has been considered on a fabricated electrode with Ir metal NPs in acidic media. The detailed mechanism for the HER for water electrolysis in acidic media is presented in ref 6. The Tafel reaction (3) is investigated for the exchange current of the electrochemical reaction on the metal iridium nanoelectrode. To get exchange current, free energy^6^ should be calculated for the Tafel reaction mechanism as follows

9 $$\Delta {G_{H}}^{*}=\Delta E+\Delta E_{\mathrm{ZPE}}-T\Delta S_{H}$$

The change of entropy Δ*S*_H_ for the Tafel reaction mechanism is estimated to be −1/2*S* ^0^H_2_ because the entropy of hydrogen on the metal surface is negligible. As a result, free energy for the HER in acidic media is written as

10 $$\Delta {G_{H}}^{*}=\Delta E+\Delta E_{\mathrm{ZPE}}+T/2^{*}\left(S^{{}^{\circ}}H_{2}\right)$$

In eq 10, Δ*E*_ZPE_ is the zero-point energy. The adsorption energy of hydrogen on the Ir metal electrode could be indicated as the following equation

11 $$\Delta E=E_{\mathrm{Ir}-H}-\left(E_{\mathrm{Ir}}+1/2H_{2}\right)$$

Δ*E*_ZPE_ is calculated based on difference-summation harmonic approximation on the vibration frequency of hydrogen adsorbed on the iridium metal surface catalyst and iridium metal catalysis. As a result

12 $$\Delta E_{\mathrm{ZPE}}=\frac{1}{2}h\sum_{i}\nu_{i}\left(\mathrm{IrH}\right)-\sum_{i}\nu_{i}\left(\mathrm{Ir}\right)$$

## 3 Results and Discussion

The catalysis material that is mentioned in the Introduction part is listed including the activity parameters for exchange current in Table 1.

**Table 1:** Catalysis List of Different Materials for the Tafel Slope as an Activity Parameter in the HER

Catalysis list	Ir in Au(111) surface	Pt support on carbon nanotube (CNT) Pt@CNT	Ir (NP)	Pt (NP)	TiNi Low content Ni 10%w in Ti	CoP (film)
Activity parameter regarding the Tafel slope in the HER	51–89 (mV/dec)^33^	11.65 (mV/dec)^32^	40–50 (mV/dec)^31^ in acid	30 (mV/dec)^30^	160 (mV/dec)^34^	42 (mV/dec)^35^

catalysis list	Ag (200 nm)	Au (bulk)	Ru@CNT	Ni–Mo alloy	Ir–Pt (nanoalloy)	Ni(OH)2
activity parameter regarding the Tafel slope in the HER	87 (mV/dec)^41^	110 (mV/dec)^40^	28.82 (mV/dec)^39^	100–134 (mV/dec)^38^	32.2 (mV/dec)^37^	30–150 (mV/dec)^36^

### 3.1 Hydrogen Adsorption Thermodynamics: Hydrogen Adsorption on Ir@SWCNT and Pt@SWCNT versus the Size of NPs, Temperature, and Pressure

Molecular dynamics (MD) simulations were performed to investigate hydrogen adsorption on iridium (Ir) and platinum (Pt) NPs supported on CNTs, representing porous electrode materials. The simulations were carried out using the DL_POLY 4.03 package.^42,43^ An NVT ensemble was employed with a total simulation time of 1 ns and a time step of 0.001 ps. The leapfrog algorithm was used to integrate Newton’s equations of motion.

The simulation cell was a cubic box with the dimensions 42.34 × 42.34 × 100.34 Å^3^, and periodic boundary conditions were applied in all directions. A single-walled carbon nanotube (SWCNT) with a diameter of 15.34 Å and a length of 40.31 Å was modeled to represent the porous electrode surface for hydrogen adsorption. The interaction cutoff in MD simulation has been taken as 10 Å. The total number of steps of MD simulation was 1,000,000 and after 800,000 steps, the system is equilibrated and data sampling is performed for the calculation of thermodynamics quantities.

The quantum Sutton–Chen (QSC) potential was employed to describe interatomic interactions among Ir atoms. This many-body potential is well-suited for modeling metallic systems and their alloys, as it accurately reproduces a variety of material properties, including lattice constants, cohesive energy, bulk modulus, elastic constants, phonon dispersion, vacancy formation energy, and surface energy.

The Lennard-Jones (LJ) potential was used to model interactions between Ir and H atoms, H_2_ molecules, and Pt and C atoms in the SWCNTs. The same LJ parameters were applied to describe Pt–H and Pt–C interactions.Molecular dynamics simulations have been extended by incorporating DFT-derived interaction potential parameters, selected for metal–hydrogen and metal–carbon interactions^44–46^ (as reported in Refs 44–46 ), to investigate hydrogen adsorption and diffusion in Ir@SWCNT, Pt@SWCNT, and Ir–Pt@SWCNT structures in the present study.

To get the optimum structure of Ir@SWCNT, Pt@SWCNT, and Ir–Pt@SWCNT, in the first step, the Ir nanocluster, Pt nanocluster, and Ir–Pt bimetallic nanocluster are optimized within the QSC potential in one nanosecond by using the NVT ensemble. In the second step, the CNT is optimized by using the Tersoff potential in one nanosecond by using the NVT ensemble. After the optimization of the metal and bimetallic nanoalloy and CNT in the first and second steps, respectively, optimization of the Ir@SWCNT, Pt@SWCNT, and Ir–Pt@SWCNT has been done with the application of the Lennard-Jones (LJ) potential between metal and carbon and QSC potential between metal atoms.

Schematic representations of Ir NPs supported on SWCNTs with varying particle sizes and hydrogen pressures are provided in the Appendix (Figures S1–S4). The results of hydrogen adsorption as a function of Ir nanoparticle (NP) size supported on carbon-based porous electrodes, using Lennard–Jones interaction potentials for Ir–carbon and Ir–hydrogen interactions, are presented in Figure 1 as Ir@SWCNT–LJ (blue). The results show that hydrogen adsorption on the Ir@SWCNT increases with the number of metal atoms in the nanoparticle, a key indicator of NP size (blue curve). Hydrogen adsorption on Pt NPs supported on nanoporous carbon electrodes was also investigated for comparison.

**Figure 1:**
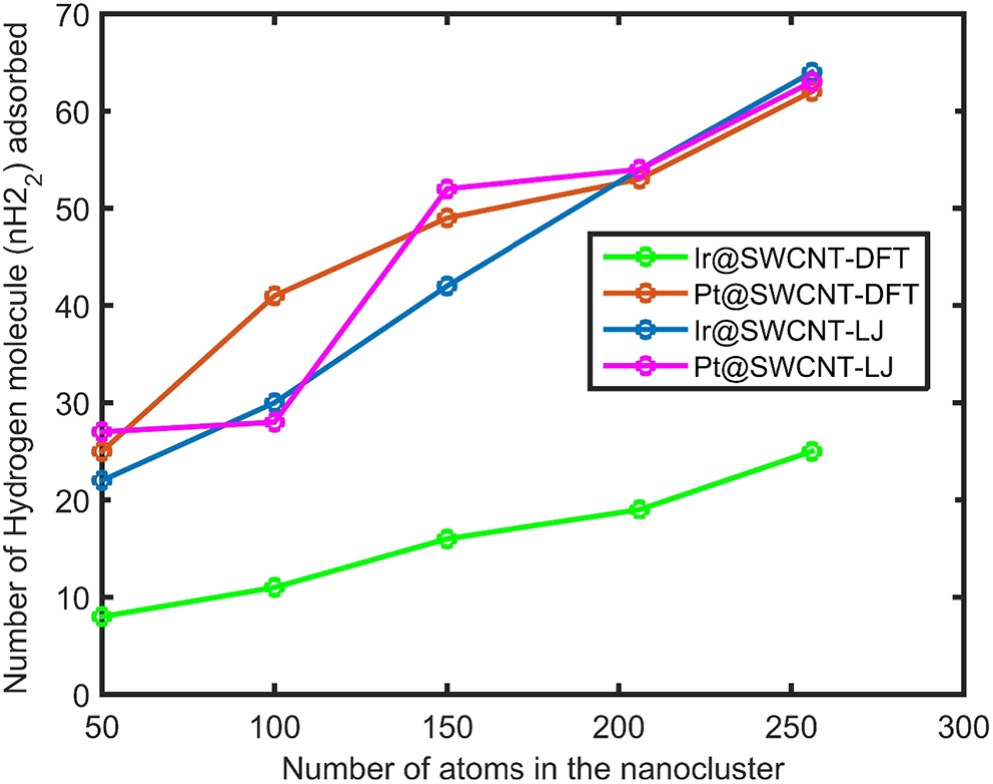
Number of hydrogen (nH_2_) molecules adsorbed on the Ir@SWCNT and Pt@SWCNT including the Lenard-Jones interaction potential between metal and carbon and metal and hydrogen atoms shown with Ir@SWCNT-LJ (blue) and Pt@SWCNT-LJ (red) versus the number of atoms in Ir and Pt nanoclusters, respectively. Hydrogen nH_2_ molecule adsorbed on the Ir@SWCNT and Pt@SWCNT by using the DFT interaction between Ir and Pt and hydrogen and carbon atoms, shown by Ir@SWCNT-DFT (green) and Pt@SWCNT-DFT (brown).

The results of hydrogen adsorption on the Pt@SWCNT, obtained using Lennard–Jones interaction potentials for Pt–carbon and Pt–hydrogen interactions, are presented in Figure 1 as Pt@SWCNT–LJ (red curve). As shown in the figure, hydrogen adsorption on platinum surfaces increases with the number of atoms in the Pt nanocluster. Physically, larger NPs possess a higher number of active sites for hydrogen adsorption, which explains the observed increase in adsorption capacity with increasing particle size. However, this trend is not strictly linear, as the surface-to-volume ratio decreases for larger particles. The number of surface atoms does not scale linearly with nanoparticle size. Several studies have investigated gas adsorption on silver and aluminum nanoparticles, as well as on silver–aluminum nanoalloy surfaces.^22,47^ The ratio of surface atoms to the total atoms in a nanoparticle varies nonlinearly with particle size.^48,49^ As a result, the number of hydrogen molecules adsorbed on the metal nanoparticle surface does not change linearly with either size or the total number of metal atoms.^22^

Molecular dynamics simulations have been extended to calculate the number of hydrogen molecules adsorbed on Ir@SWCNT and Pt@SWCNT systems based on DFT-derived interaction potentials for metal–hydrogen and metal–carbon interactions. The results of hydrogen adsorption as a function of the number of Ir and Pt atoms in Ir@SWCNT and Pt@SWCNT systems, obtained using these DFT-based potentials, are presented in Figure 1 as Ir@SWCNT–DFT (green) and Pt@SWCNT–DFT (brown), respectively. The DFT-based interaction potentials indicate that hydrogen adsorption increases with the increasing number of metal atoms.

For Ir@SWCNT, the number of adsorbed hydrogen molecules predicted using the DFT interaction potential is lower than that obtained from the Lennard-Jones (LJ) potential. In the case of Pt@SWCNT, for systems with fewer than 150 Pt atoms, the DFT results predict higher hydrogen adsorption than the LJ potential. However, for larger systems containing more than 150 Pt atoms, the hydrogen adsorption predicted by the LJ potential between metal–hydrogen and metal–carbon interactions exceeds that obtained from the DFT-based interaction potential. A stronger interaction potential depth for metal–hydrogen in the Lennard-Jones (LJ) model compared to the DFT-based interaction potential leads to higher hydrogen adsorption predicted by the LJ interaction model.

For smaller Pt nanoclusters (∼50–100 atoms), Pt@SWCNT exhibits slightly higher hydrogen adsorption capacity than that of Ir@SWCNT, whereas for intermediate Ir nanocluster sizes (∼100–150 atoms), Ir@SWCNT shows greater adsorption. This suggests that hydrogen adsorption is sensitive to the electronic structure and surface chemistry of the metal NP. At larger numbers of atoms in the nanocluster (∼200–250), both Ir and Pt display nearly identical hydrogen adsorption behavior, indicating that at these scales, the dominant factor governing adsorption is the available surface area rather than the specific metal type.

Hydrogen adsorption as a function of temperature was also investigated on the surface of an iridium (Ir) NP. The simulation results for hydrogen adsorption on Ir supported on carbon nanotubes as a fabricated electrode are presented in Figure S5. As shown, hydrogen adsorption decreases monotonically with an increase in temperature. This behavior can be attributed to the increase in the kinetic energy at higher temperatures, which facilitates the desorption of hydrogen molecules from the Ir nanocatalyst surface.

Similarly, hydrogen adsorption on Pt@SWCNT was examined across a range of temperatures, with the results being presented in Figure S6. As in the case of Ir, hydrogen adsorption on Pt NPs also decreases with temperature, exhibiting a monotonic trend. This indicates that thermal effects consistently weaken the interaction between hydrogen molecules and metal nanoparticle surfaces.

A comparison of hydrogen adsorption as a function of temperature on Pt@SWCNT and Ir@SWCNT when the number of metal atoms in the nanocluster is *N* = 256 is shown in Figure 2. At lower temperatures (300 K < *T* < 330 K), hydrogen adsorption on the Ir NP is higher than that on the Pt NP. However, at higher temperatures (320 K < *T* < 350 K), the Pt NP exhibits greater hydrogen adsorption than that of the Ir NP. In this temperature range, the kinetic energy of hydrogen molecules can overcome the potential adsorption energy on Ir surfaces, leading to hydrogen desorption from the Ir@SWCNT electrode. In contrast, due to the stronger hydrogen–metal interaction on Pt, desorption from Pt@SWCNT is less favorable, resulting in a higher hydrogen adsorption capacity compared to Ir@SWCNT at elevated temperatures.

**Figure 2:**
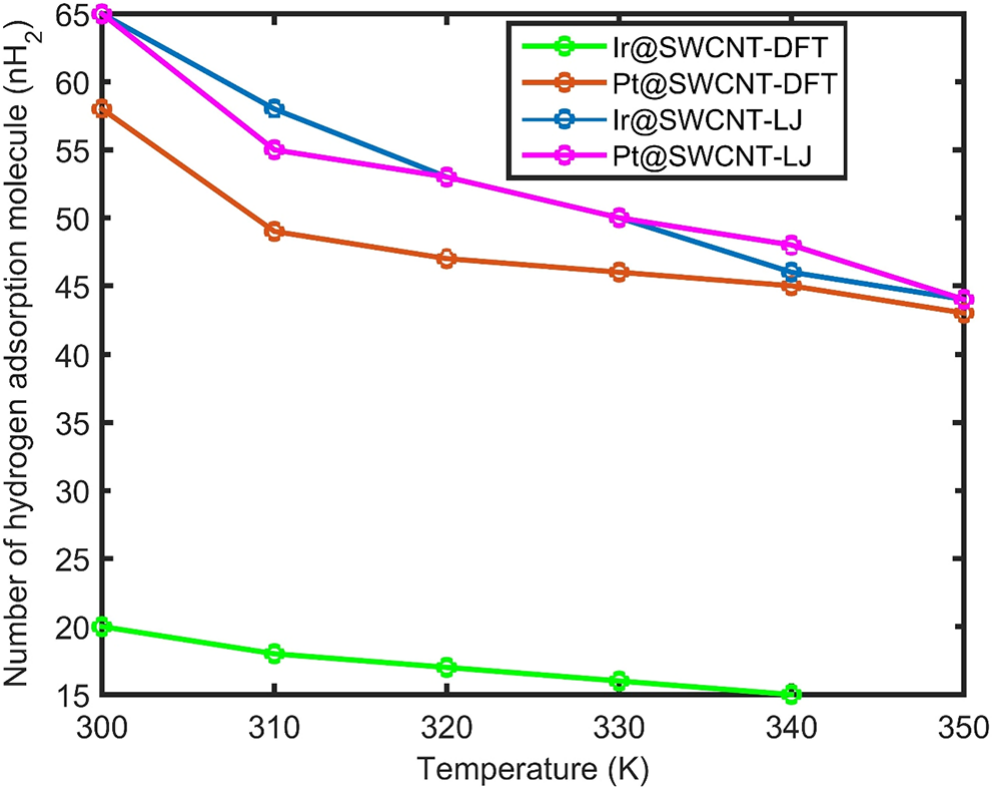
Comparison of the number of adsorbed hydrogen molecules (nH_2_) for Ir@SWCNT (blue) and Pt @SWCNT (red) versus temperature based on the LJ interaction potential between metal and hydrogen and metal and carbon.

Hydrogen adsorption via DFT potential parametrization between metal and hydrogen and metal and carbon on Ir@SWCNT (green) and Pt @SWCNT (brown) is shown. Results for hydrogen adsorption via the LJ potential and DFT interaction potential on Ir and Pt nanoclusters supported by SWCNTs are shown as Ir@SWCNT-LJ, Pt @SWCNT-LJ, Ir@SWCNT-DFT, and Pt @SWCNT-DFT, respectively.

Molecular dynamics simulations have been extended using DFT-derived interaction potentials for metal–hydrogen and metal–carbon interactions to study hydrogen adsorption as a function of temperature on Ir@SWCNT and Pt@SWCNT systems. The DFT-based results show that hydrogen adsorption on both Ir@SWCNT and Pt@SWCNT decreases monotonically with increasing temperature. In the temperature range of 300 K ≤ T ≤ 350 K, the number of hydrogen molecules predicted using the DFT interaction potential is lower than that obtained using the Lennard-Jones (LJ) potential for both metal–hydrogen and metal–carbon interactions.

The hydrogen adsorption behavior of Ir@SWCNT was analyzed as a function of pressure, with the results presented in Figure 3 (blue curve). As shown, hydrogen adsorption on the surface of an iridium NP increases with increasing pressure. This trend can be attributed to the greater availability of hydrogen molecules at higher pressures, which enhances their probability of adsorption on the Ir nanocatalyst’s surface. Consequently, hydrogen adsorption increases proportionally with the pressure on the metallic NP surface.

**Figure 3:**
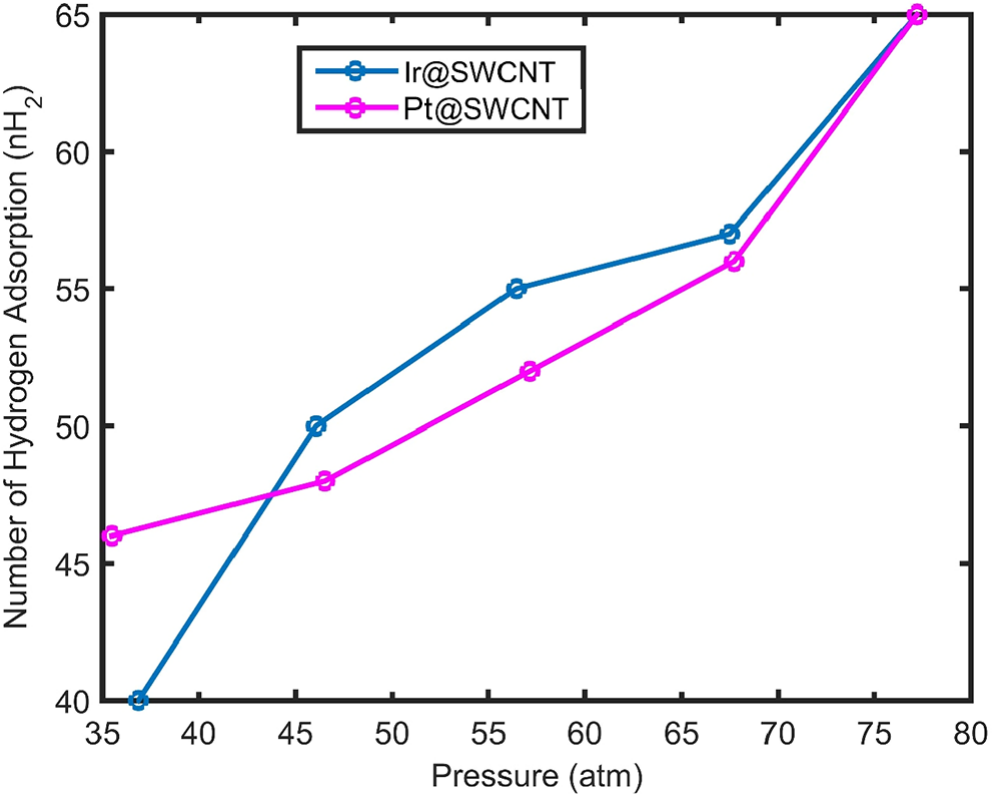
Number of adsorbed hydrogen molecules (nH_2_) on Ir@SWCNT versus pressure (blue) and number of adsorbed hydrogen molecules (nH_2_) for Pt@SWCNT versus pressure (red).

The pressure effect has been considered on hydrogen adsorption on Pt@SWCNT. The result of hydrogen adsorption versus pressure on the Pt nanoparticle surface is presented in Figure 3 (red). At high hydrogen gas pressure, more hydrogen molecules adsorb on the surface of the Pt@SWCNT. However, according to Figure 3, at high pressures, Ir@SWCNT stores more hydrogen molecules than Pt@SWCNT. This indicates that Ir@SWCNT has a greater number of available surface adsorption sites compared with Pt@SWCNT. Therefore, with an increase in hydrogen pressure at constant room temperature and volume, more hydrogen molecules are able to occupy the relatively more abundant adsorption sites on Ir@SWCNT than on Pt@SWCNT.

### 3.2 Hydrogen Adsorption on the Ir–Pt Nanoalloy Supported on Carbon Nanotubes

Hydrogen adsorption on Pt–Ir nanoalloys supported on single-walled carbon nanotubes (SWCNTs) was investigated as a function of the Pt doping level. Schematic representations of the simulation setups for hydrogen adsorption on Pt–Ir nanocatalysts with varying Pt contents (20%, 50%, 80%, and 100%) supported on SWCNTs are shown in Figure S7A–D, respectively.

The corresponding hydrogen adsorption results for Ir–Pt@SWCNT as a function of Pt composition, including Lennard-Jones (LJ) interactions between metal and hydrogen and metal and carbon, are presented in Figure 4 (denoted as Ir–Pt@SWCNT–LJ, shown in brown). The results indicate that a lower Pt content in the Pt–Ir nanoalloy leads to a higher hydrogen adsorption capacity. This behavior suggests that moderate Pt doping enhances the adsorption performance of Ir-based nanocatalysts supported on SWCNTs. Based on the LJ interaction between metal and hydrogen and metal and carbon, the incorporation of a small amount of Pt into Ir@SWCNT induces the migration of some Ir atoms from the nanoparticle core toward the surface, where they interact with Pt atoms. This atomic rearrangement increases the number of surface atoms in the Ir–Pt@SWCNT structure, thereby providing more active sites for hydrogen adsorption. Consequently, the hydrogen adsorption capacity improves due to the formation of additional adsorption sites. Molecular dynamics simulations have been extended to investigate hydrogen adsorption on Ir–Pt@SWCNTs, in comparison with Pt doping, using DFT-derived interaction potentials between Ir and Pt atoms and hydrogen and carbon to achieve more accurate results. The hydrogen adsorption results based on DFT-parametrized potentials for the Ir–Pt@SWCNT system are also shown in Figure 4 (denoted as Ir–Pt@SWCNT–DFT, shown in blue). According to Figure 4, the hydrogen adsorption predicted using DFT-based potentials is lower than that obtained using LJ interactions. For Pt_*x*_ Ir_1–*x*_@SWCNT compositions with *x* ≤ 0.5, the trends in hydrogen adsorption predicted by DFT and LJ potentials are opposite. However, for compositions with *x* > 0.5, both DFT and LJ approaches exhibit similar trends in hydrogen adsorption as a function of the Pt content.

**Figure 4:**
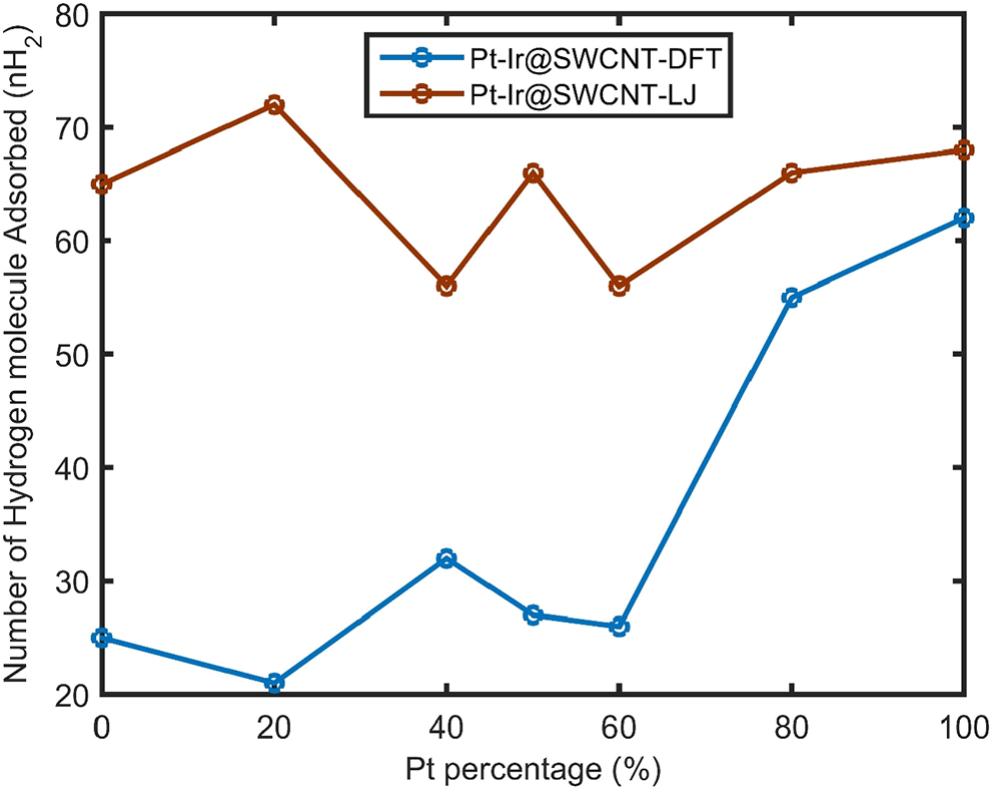
Number of hydrogen molecules (*n*H_2_) adsorbed on Pt_*x*_ Ir_1–*x*_ @SWCNT versus Pt doping (*x* variable), in 256 total atoms in the Pt–Ir nanocluster via the Lenard-Jones interaction between metal and hydrogen and metal and carbon, shown in brown as Pt_*x*_ Ir_1–*x*_ @SWCNT-LJ. Number of hydrogen molecules (*n*H_2_) adsorbed on Pt_*x*_ Ir_1–*x*_ @SWCNT versus Pt doping (*x* variable), in 256 total atoms in the Pt–Ir nanocluster via the DFT potential interaction between metal and hydrogen and metal and carbon, shown in blue as Pt_*x*_ Ir_1–*x*_ @SWCNT-DFT.

Surface coverage of hydrogen on Ir@SWCNT versus temperature is presented in Figure S8. According to Figure S8, surface coverage of Ir@SWCNT decreases versus temperature generally.

### 3.3 Temperature Dependency of Heat Capacity for Hydrogen Adsorption for Large Ir NPs Supported on Carbon Nanotubes

Heat capacity for hydrogen adsorption on Ir@SWCNT as a porous electrode surface has been considered versus temperature. For the calculation of heat capacity versus temperature, the following process could be imagined

13 $$M+\frac{1}{2}H_{\mathrm{2(}g)}\to {\mathrm{MH}}_{\left(g\right)}$$

The enthalpy of the process could be defined as follows

14 $$H_{\mathrm{process}}=E_{\mathrm{procss}}+{\mathrm{PV}}_{\mathrm{process}}$$

In eq 14, the variables *H*, *E*, *P*, and *V* represent the enthalpy of the process, the energy of the process, the system pressure, and the system volume, respectively.

The change in the energy of the process, as given in eq 15, can be written as

15 $$\Delta E_{\mathrm{process}}=E_{\left(M-H\right)s}-\left(E_{\left(M\right)s}+1/2H_{2}\right)$$

*E*_(M‑H)s_ and *E*_(M)s_ are the energies of metal hydrogen and metal that are supported on carbon nanotubes, respectively.

The change of enthalpy for hydrogen adsorption on Ir@SWCNT could be written as

16 $$\Delta H_{\mathrm{process}}=\Delta E_{\mathrm{process}}+P\Delta V_{\mathrm{process}}$$

In eq 16, the change in energy is expressed using eq 15, and the ideal gas assumption is applied to calculate the change in volume during the process. It is assumed that the change in volume is approximately proportional to the volume of hydrogen. The heat capacity as a function of temperature for Ir nanoparticles with larger nanocluster sizes (100 and 256 atoms), supported on single-walled carbon nanotubes (SWCNTs) as porous electrodes, is presented in Figure 5A,B, respectively. For these relatively large Ir nanoparticles, a nonmonotonic dependence of heat capacity on temperature is observed.

**Figure 5:**
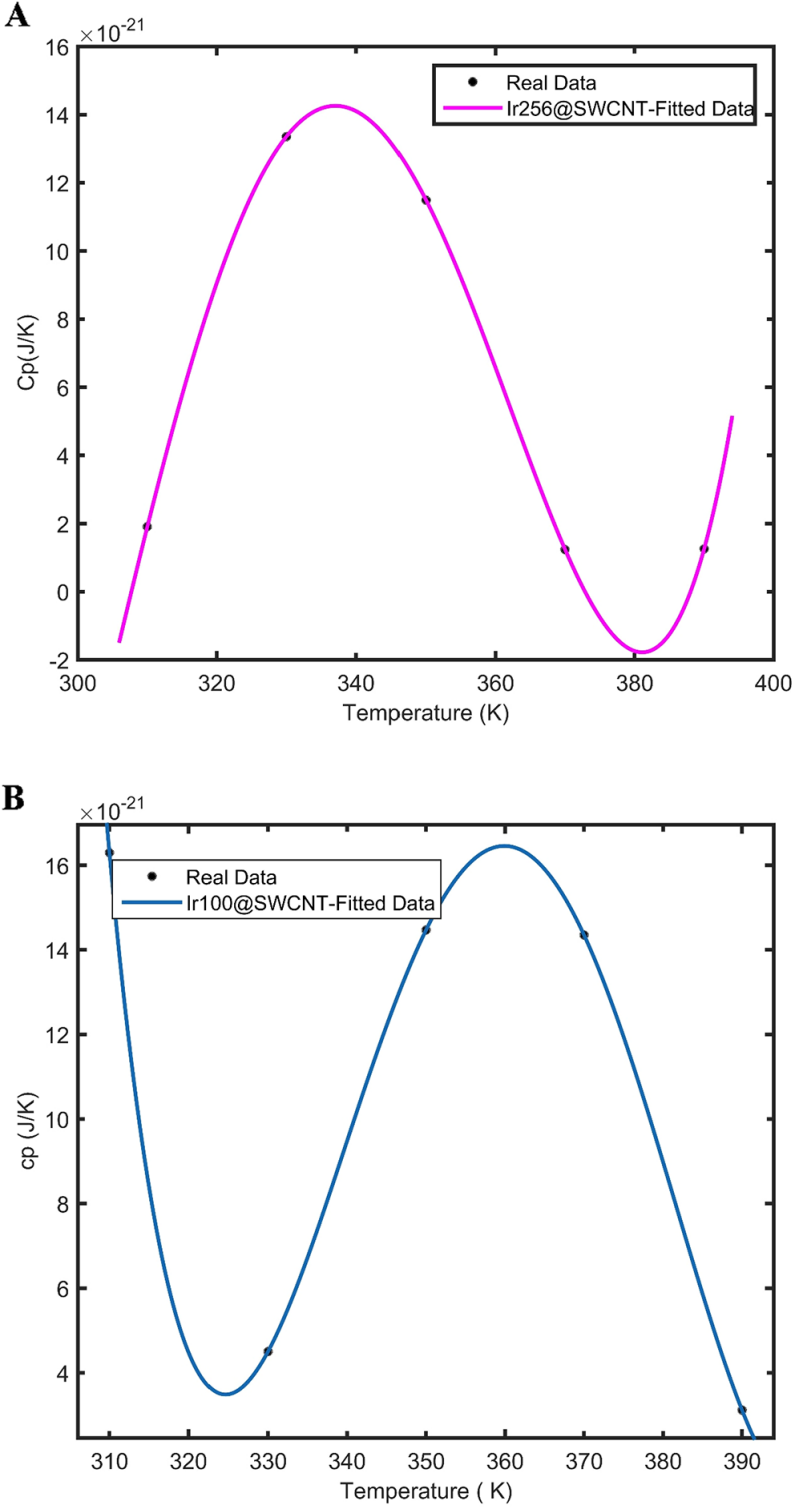
(A) Nonmonotonic behavior of heat capacity (Joule per Kelvin (J/K)) versus temperature for an Ir nanocluster with 256 atoms supported on a carbon nanotube. Black points represent real simulation data, and the solid line represents the fitting result. (B) Nonmonotonic behavior of heat capacity (J/K) versus temperature for an Ir nanocluster with a total atom number of 100. Black points represent real simulation data, and the solid line represents the fitting result.

As shown in Figure 6, the energy levels for Ir NPs supported on carbon nanotubes (the number of atoms in the nanocluster being 100 and 256) can be represented across four temperature points (*T*_0_, *T*_1_, *T*_2_, *T*_3_). To explain the heat capacity, the canonical ensemble can be assumed for describing the physical system. In the canonical ensemble, the heat capacity (*C*_p_) is defined as the derivative of energy with respect to temperature, or alternatively, it can be expressed in terms of energy fluctuations within the system. Fluctuation energy for *T*_1_ is very small or all particles arranged in one energy level. Assuming equal temperature changes between successive temperature intervals (*T*_0_ → *T*_1_, *T*_1_ → *T*_2_, *T*_2_ → *T*_3_), the variation of heat capacity can be explained as follows:

- For Ir_100_@SWCNT at very low temperature *T*_0_, particles occupy the lowest-energy level *E*_1_ (Figure 6A).
- At *T*_1_, the distribution changes, giving rise to a corresponding heat capacity of Cp_1_ (Figure 6B).
- As the temperature increases to *T*_2_, another distribution appears, resulting in Cp_2_ (Figure 6C).
- Finally, at *T*_3_, the distribution differs again, yielding a new heat capacity value of Cp_3_ (Figure 6D).

**Figure 6:**
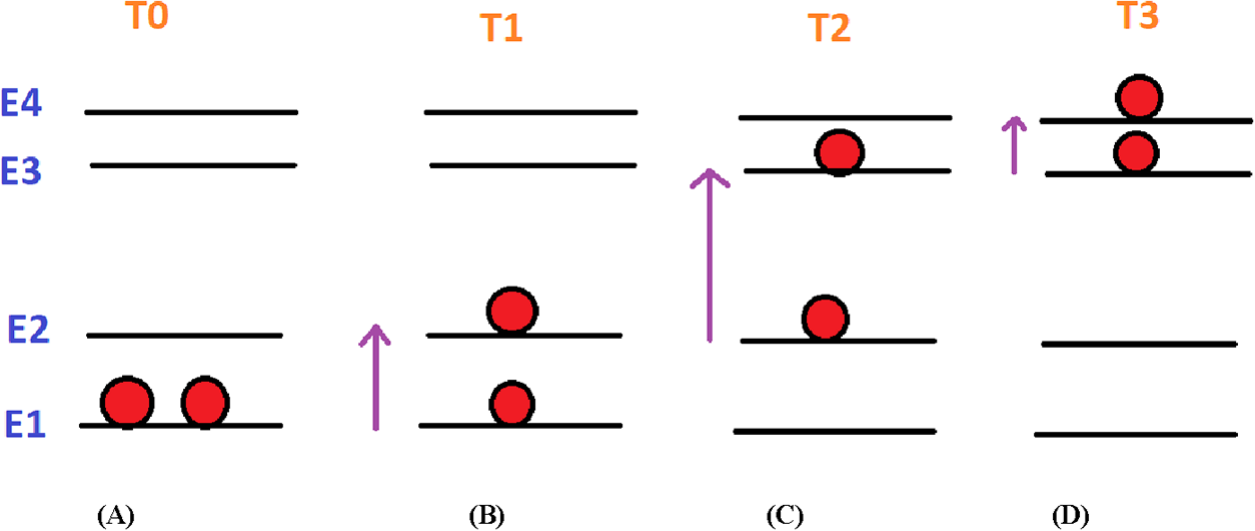
Imagination for the energy diagram for an Ir nanocluster with an atom number of 100 supported on the SWCNT (Ir_100_@SWCNT) and Ir nanocluster with an atom number of 256 supported on the SWCNT (Ir_256_@SWCNT) with particle distribution at temperatures of (A) *T*_0_, (B) *T*_1_, (C) *T*_2_, and (D) *T*_3_.

The resulting trend for Ir_100_@SWCNT is nonmonotonic: Cp_1_ < Cp_2_, Cp_3_ < Cp_2_. A similar explanation applies to the Ir_256_@SWCNT.

### 3.4 Temperature Dependency of Heat capacity for Hydrogen Adsorption on Small-Sized Ir NPs Supported on Carbon Nanotubes

The results of heat capacity versus temperature for Ir nanoparticles with a small number of atoms in the nanocluster (50), supported on single-walled carbon nanotubes (SWCNTs) as porous electrodes, are presented in Figure 7. According to Figure 7, the heat capacity of Ir@SWCNT exhibits monotonic behavior as a function of temperature for a small number of Ir atoms (50 atoms), supported on the SWCNT.

**Figure 7:**
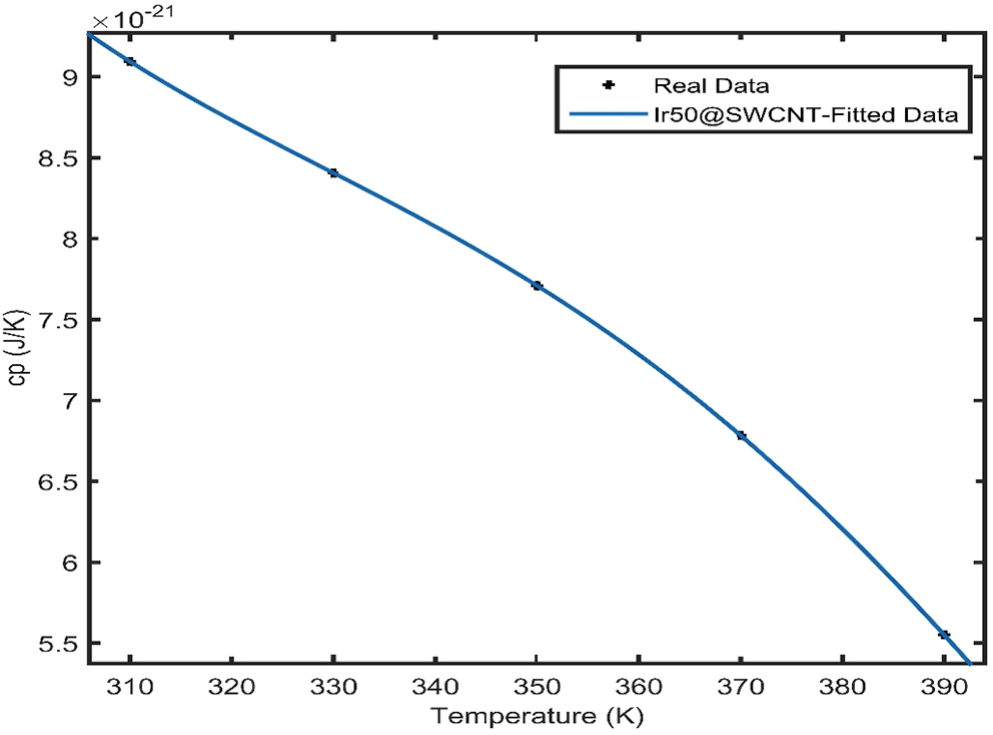
Monotonic behavior for heat capacity versus temperature for Ir NPs with a total atom number of 50 supported on the carbon nanotube.

The heat capacity shown in Figure 7 was fitted to a power series up to the fourth order of the temperature variable. The results of this fitting are presented in Table 2S in the Supporting Information.

Our calculation of the melting temperature of pure iridium in the face-centered cubic bulk structure is 2716 K, which is in agreement with the available experimental data.^50^ In Figure 7, the black data points represent the results obtained from molecular dynamics simulations, while the solid lines correspond to the fourth-order algebraic fits performed by using the MATLAB curve-fitting toolbox for heat capacity versus temperature. The observed monotonic and nonmonotonic behaviors of heat capacity with temperature are related to the distribution of energy levels in Ir nanoparticles of different sizes supported on carbon nanomaterials. Specifically, small Ir nanoparticles supported on carbon nanoporous materials exhibit different energy level distributions compared with larger Ir nanoparticles supported on carbon nanotubes.

In contrast, Figure 8 shows the energy diagram and particle distributions for a small number of Ir atoms in Ir nanoparticles (Ir_50_@SWCNT) across the same four temperature points. In this case, as the temperature increases from *T*_1_ to *T*_2_ and then from *T*_2_ to *T*_3_, the heat capacity decreases continuously. Thus, the heat capacity behavior of Ir_50_@SWCNT decreases monotonically with the temperature.

**Figure 8:**
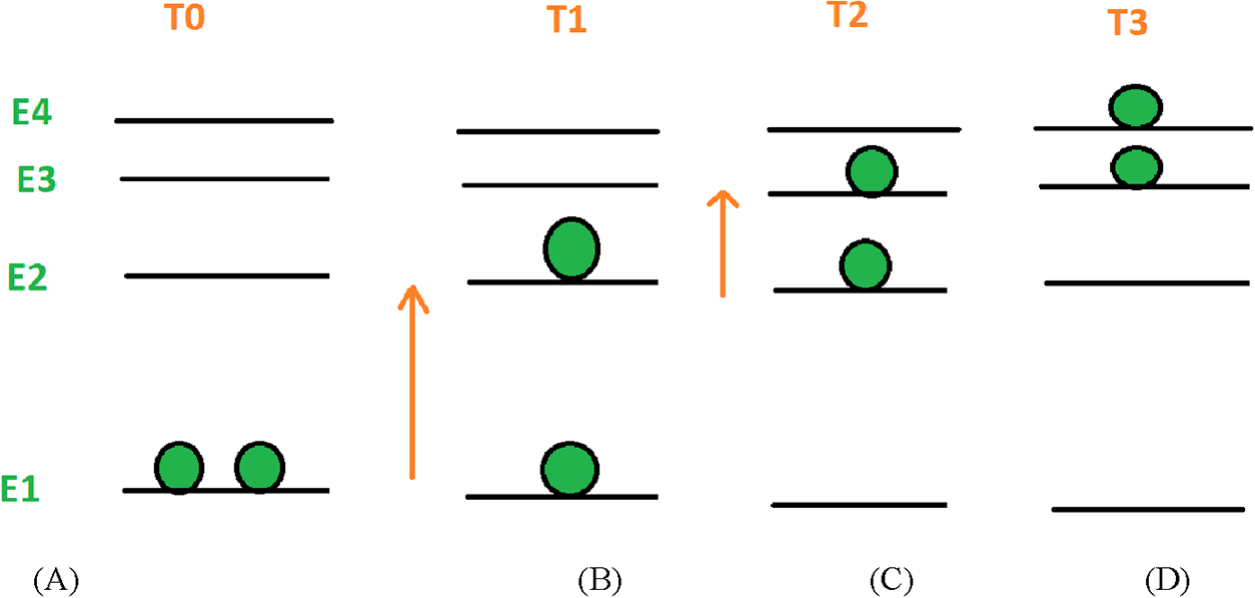
Imagination for the energy diagram for an Ir nanocluster with a total atom number of 50 supported on the SWCNT (Ir_50_@SWCNT) with particle distribution at temperatures of (A) *T*_0_, (B) *T*_1_, (C) *T*_2_, and (D) *T*_3_.

### 3.5 Hydrogen Diffusion behavior on Ir@SWCNT and Pt@SWCNT versus the Number of Atoms in the Nanocluster

The diffusion of hydrogen on Ir@SWCNT is calculated with the following equation

17 $$D\equiv \frac{1}{2d}\lim_{t\to \infty }\frac{\left\langle {\left[r\left(t_{0}+t\right)-r\left(t_{0}\right)\right]}^{2}\right\rangle }{t}$$

where *D*, *d*, *r*(*t*_0_+*t*), and *r* (*t*_0_) are the diffusion coefficient, dimensionality of the system, and the vector position of atoms at time (*t*) and zero (*t*_0_), respectively.

The diffusion coefficient of hydrogen gas on iridium nanoparticles supported on carbon porous electrodes is presented in Figure 8 (blue curve). The results show that hydrogen diffusion is significantly higher for smaller iridium nanocatalysts supported on carbon nanotubes acting as porous electrodes. Overall, the hydrogen diffusion coefficient on Ir@SWCNT is greater than that on Pt@SWCNT in the smaller particle size range.

Hydrogen diffusion versus the number of atoms in the metallic nanoparticle based on the DFT potential interaction between metal and hydrogen and metal and carbon in Ir@SWCNT and Pt@SWCNT is denoted with Ir@SWCNT-DFT (green) and Pt@SWCNT-DFT (brown), respectively.

The results of hydrogen diffusion on Pt@SWCNT are shown in Figure 9 (red curve). According to eq 17, the diffusion coefficient of a hydrogen molecule on a metal-supported carbon nanotube surface is proportional to the standard deviation of the hydrogen molecule’s position over time. Due to the stronger interaction of hydrogen molecules with Pt surface sites compared to Ir atoms, hydrogen molecules tend to be more localized on Pt surfaces. This stronger interaction leads to greater localization and, consequently, a smaller diffusion coefficient. As a result, the diffusion coefficient of hydrogen on Pt@SWCNT is lower than that on Ir@SWCNT. Molecular dynamics simulations have been extended to calculate hydrogen diffusion as a function of the number of metal atoms (i.e., nanoparticle size) in Ir@SWCNT and Pt@SWCNT systems, using DFT-derived interaction potentials for metal–hydrogen and metal–carbon interactions. The results for hydrogen diffusion versus the number of metal atoms are presented in Figure 9, where Ir@SWCNT–DFT and Pt@SWCNT–DFT are denoted by green and brown symbols, respectively. As shown in Figure 9, hydrogen diffusion based on DFT interaction potentials is higher than that obtained using Lennard-Jones (LJ) potentials for both systems. This can be attributed to the deeper potential well of the LJ interaction for metal–hydrogen compared to the DFT-derived potential. Consequently, the stronger interaction in the LJ model restricts the mobility of hydrogen atoms, reducing their freedom to move and limiting their dispersion over time. In contrast, the weaker interaction described by the DFT potential allows greater mobility, leading to higher hydrogen diffusion.

**Figure 9:**
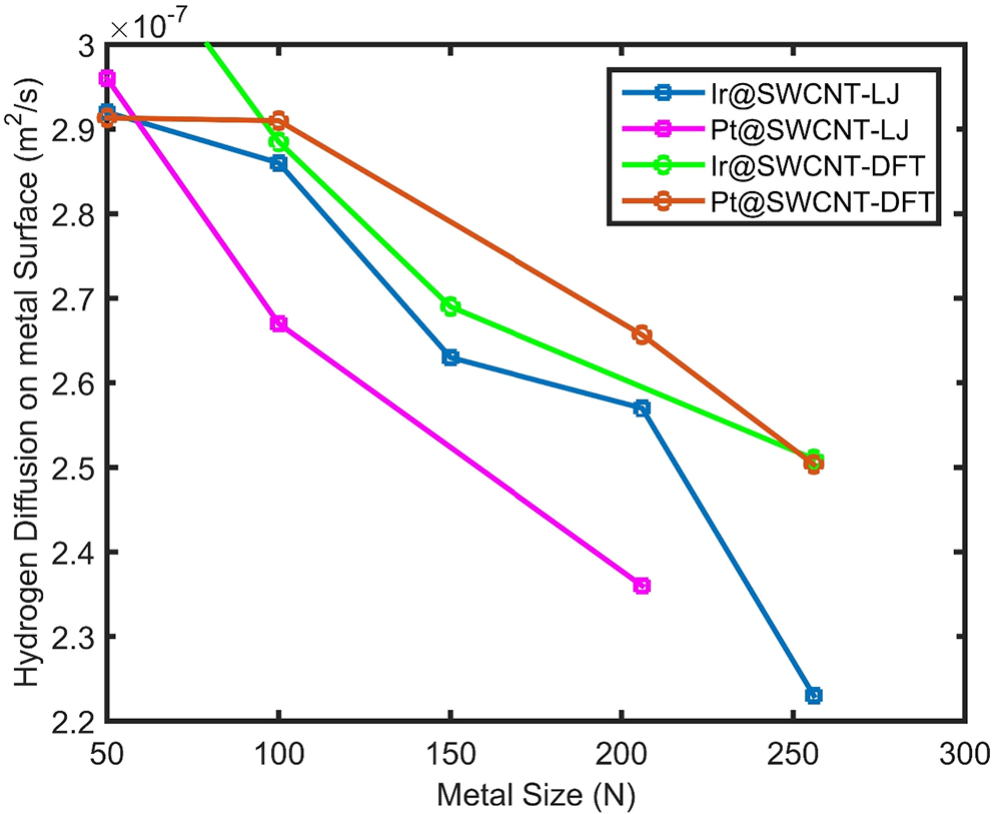
Hydrogen diffusion on Ir@SWCNT versus the number of Ir atoms in the Ir nanocluster (blue) and hydrogen diffusion on Pt@SWCNT versus the number of platinum atoms in the Pt nanocluster (red) based on the Lenard-Jones potential between metal and hydrogen and metal and carbon, shown as Ir@SWCNT-LJ and Pt@SWCNT-LJ, respectively.

## 4 HER in Water Electrolysis

### 4.1 Vibration Spectroscopy for the HER in Water Electrolysis on the Ir Nanoparticle Surface

Density functional theory (DFT) calculations were performed to analyze the vibrational density associated with the hydrogen evolution reaction (HER) during water electrolysis. The adsorption intensity fraction for the vibrational spectrum for chemisorbed hydrogen on the surface of an icosahedral Ir NP (representing the Tafel step, eq 3) is presented in Figure 10A. Two distinct vibrational peaks are observed, corresponding to the formation of chemical bonds between hydrogen atoms and the Ir surface. These results, obtained for a 13-atom icosahedron Ir nanoparticle, highlight its active role as a nanocatalyst in the HER.

**Figure 10:**
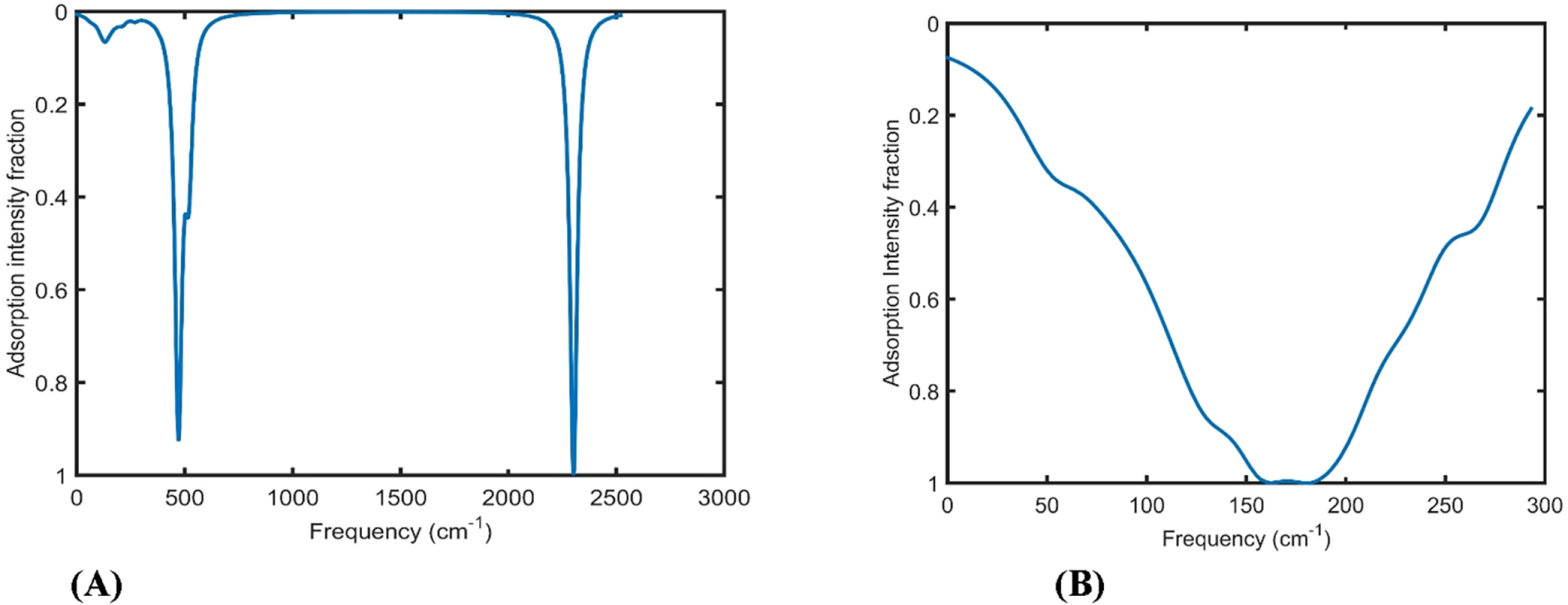
(A) Adsorption intensity fraction for the vibrational spectrum regarding hydrogen adsorption on the Ir metal nanocluster with an Ir total atom number of 13 with an icosahedron structure. (B) Adsorption intensity fraction for the vibrational spectrum versus frequency (cm^–1^) of the Ir metal nanocluster with an Ir total atom number of 13 for the HER in water electrolysis.

Also, the adsorption intensity fraction for the vibrational spectrum for the icosahedron Ir nanocluster with 13 atoms is presented in Figure 10B. The result of vibration spectroscopy in Figure 10B shows one extended wide peak for the Ir icosahedron with a total atom number of 13. For a small-sized Ir nanocluster, there is no such difference between the core and surface atoms due to the very small size. As a result, the phonon density of core and surface atoms appears as one wide peak. Based on Figure 10A, when hydrogen adsorbs on the metal surface, it forms a new chemical bond, and a main peak in the vibrational spectrum density corresponding to the metal–hydrogen (Ir–H) bond is observed at a very high frequency of around 2300 cm^–1^. Figure 10A also shows a peak at a lower frequency (470 cm^–1^) in the vibrational spectrum density for the Tafel step (eq 3) in the Ir nanocluster structure. Furthermore, a frequency shift is observed for the broad vibrational spectrum density peak at 175 cm^–1^ for the pure Ir cluster in Figure 10B. The physical origin of the 470 cm^–1^ peak in Figure 10A is the structural change of the Ir nanocluster induced by hydrogen adsorption, which shortens the chemical distance between two Ir atoms and results in the appearance of this higher frequency adsorption peak in the vibrational spectrum density.

The peak position and intensity can be correlated with the catalytic activity and the efficiency of hydrogen production. At lower vibrational frequencies of the metal–hydrogen (M–H) bond, less energy is required to break the Ir–H bond, indicating that Ir nanoparticles exhibit favorable catalytic activity for hydrogen production. Additionally, the higher peak intensity of the Ir–H bond suggests that a greater amount of hydrogen can be released during the hydrogen evolution reaction (HER), particularly in the Tafel step.

### 4.2 Tafel Exchange Current in the HER on Ir@SWCNT

The Tafel exchange current has been considered for the HER on Ir@SWCNT. The Tafel exchange current for the HER in acidic media is calculated based on the free energy of hydrogen adsorption in the metal surface.

To investigate the hydrogen adsorption process on iridium metal nanoparticles, density functional theory (DFT) calculations were performed using the Quantum ESPRESSO package. The study focused on evaluating the free energy as a function of Ir NP size for the hydrogen evolution reaction (HER) for the Tafel step in water electrolysis. For calculation of the free energy in the Tafel step for the HER, eqs 9–12 have been used, and the main contribution of entropy for the Tafel step reaction mechanism is that of a hydrogen molecule in the gas phase approximately because of the following equation. The entropy of a hydrogen molecule in the gas phase at room temperature is around 0.4 eV.

18 $$\mathrm{MH}\left(\mathrm{ads}\right)+\mathrm{MH}\left(\mathrm{ads}\right)⇌H_{2\left(g\right)}+2M,\Delta S_{\mathrm{Tafel Step}}=\left(\Delta S_{H_{2}}+2\Delta S_{M}\right)-2\Delta S_{M-H}≃\Delta S_{H_{2}\left(g\right)}$$

The zero-point energy for hydrogen adsorption on an Ir metal nanoparticle (eq 12) is 0.04 eV.

The calculations employed ultrasoft pseudopotentials and the Perdew–Burke–Ernzerhof (PBE) exchange-correlation functional. An energy cutoff of 40 Ry was used for the plane-wave basis set describing the wave functions, and 400 Ry was used for the charge density. All calculations were conducted using the γ-point.

The result of free energy versus the number of atoms in the Ir nanoparticle for the Tafel step HER in water electrolysis (eq 3) is presented in Figure 11A. According to Figure 11A, there is nonmonotonic behavior for the free energy of the HER on the Ir nanocatalyst surface versus size. The maximum free energy for the HER in the Tafel step reaction (eq 3) during water electrolysis is associated with the amorphous Ir structure. When hydrogen adsorbs on the amorphous structure, it induces strong stress within the Ir nanocluster, while the interaction between metal and hydrogen remains relatively weak. As a result, the free energy for the amorphous phase in the Tafel step is higher than those of other Ir nanostructures. Therefore, a nonmonotonic behavior of free energy is observed for the Tafel step reaction when transitioning from regular Ir nanocluster shapes to the amorphous phase. The result of Tafel exchange current by using equation 19 is presented in Figure 11B. Based on the previous litrature,^6^ *k*_0_ in eq 19 has been included as 200/s for the calculation of the Tafel exchange current. Δ*G* in eq 19 is the free energy of hydrogen adsorption on the metal surface in the Tafel step for the HER in water electrolysis.

**Figure 11:**
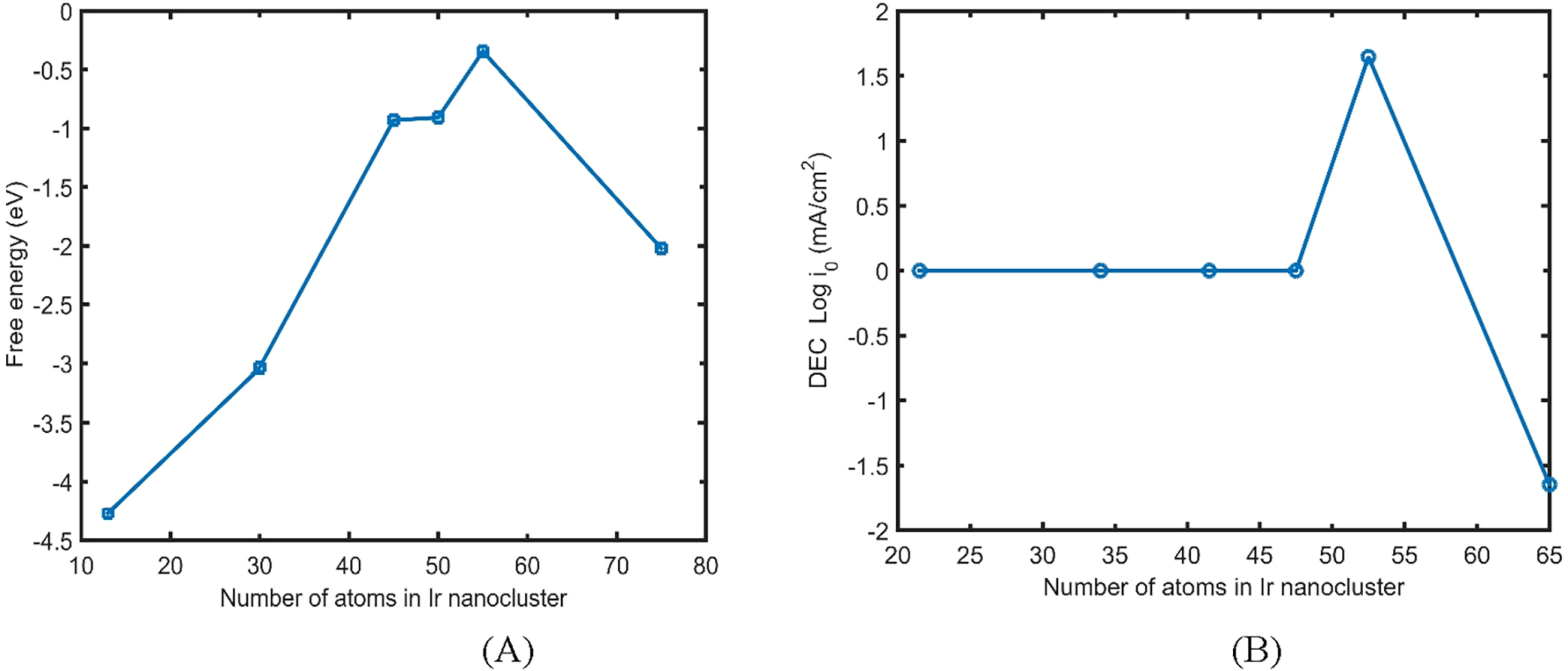
(A) Free energy electronvolt (eV) of Ir nanocluster versus the number of Ir atoms in the Ir nanocluster. (B) Log of difference exchange current (DEC) versus the number of Ir atom in the iridium nanocluster.

19 $$I_{0}=k_{0}\;\exp\left(\frac{-\Delta G}{\mathrm{RT}}\right)$$

The difference exchange current (DEC) between two consecutive NPs in different atoms (namely, *I*_N(i+1)_ – *I*_N(i)_) as a function of Ir nanoparticle atoms is presented in Figure 11B. According to Figure 10B, the maximum difference in exchange current is associated with the amorphous structure of the NP. Figure 11B further reveals a nonmonotonic trend in the exchange current with respect to particle size for the hydrogen evolution reaction (HER) on Ir nanocatalysts.

Density functional theory (DFT) calculations indicate that small iridium nanoparticles (20–50 atoms, ∼1 nm in size) exhibit an exchange current density of approximately 1.1 mA cm^–2^ for the hydrogen evolution reaction (HER) in acidic media, which increases to nearly 5 mA cm^–2^ upon undergoing an amorphous phase transition. Experimental results^51^ for an Ir nanoelectrode with a size of 40 nm report an exchange current density of about 1.3 mA cm^–2^ under similar conditions. Thus, increasing the Ir nanoparticle size from ∼1 to 40 nm leads to a relatively modest increase in exchange current density, from approximately 1.1 to 1.3 mA cm^–2^.

Based on eq 19, the free energy in the Tafel step reaction makes a significant contribution to the exchange current for the HER in water electrolysis. The sharp increase in exchange current observed in Figure 11B is associated with the corresponding jump in free energy when transitioning from regular Ir nanocluster shapes to the amorphous phase. Therefore, a discontinuity in the exchange current for the HER is expected during the structural transformation from crystalline Ir nanoclusters to the amorphous phase. The order of exchange current density for the Ir nanoparticle, which is presented in Figure 11B, is in agreement with the experimental values of Ir in acidic HER conditions, which are on the order of 0.1–1 mA/cm^2^, but this can vary depending on the nanoparticle size, support, and synthesis method.^51,52^ It is worthwhile to notice that solvent, potential, and double-layer effects are neglected in the DFT calculations.

### 4.3 Current Density for Ir, Pt, and the Ir–Pt Nanoalloy for the HER in Water Electrolysis in Alkaline Media

The electrical current density for water electrolysis on Ir–Pt nanoalloys in alkaline solution was calculated using eq 7. Density functional theory (DFT) was employed to evaluate the forward and backward chemical reaction rates for reactions 1–3. The backward reaction rates were obtained from Gibbs free energy calculations using the Quantum ESPRESSO package, which employed ultrasoft pseudopotentials, the Perdew–Burke–Ernzerhof exchange-correlation functional, an energy cutoff of 40 Ry for the plane-wave basis set, 400 Ry for the electronic density (1 Ry = 13.606 eV), and ∼8 Å of vacuum between replicated cells. Brillouin zone sampling was performed at the γ-point, and all calculations were spin-unrestricted. The nudged elastic band (NEB) approach has been used for the calculation of vacancy rate and vacancy diffusion within the silver nanocluster and free energy calculation for the HER in different metal surfaces.^53,54^ The nudged elastic band (NEB) approach has been used for the calculation of chemical rate of forward reactions in HER eqs 1–3. This method searches for the minimum energy path between two local minima by creating a fixed number of intermediate configurations (images) that are linked to each other by elastic springs. The image highest in energy does not feel the spring forces along the band; instead, the true force at this image along the tangent is inverted. In this way, the image tries to maximize its energy along the band, and thus, when this image converges, it is at the exact saddle point. The local minima singled out by a preliminary structural search are set as the starting configuration NEB procedure, using a number of intermediate images ranging from 3 to 7. The Gibbs free energy for all chemical reactions 1–3 for the HER in alkaline media via the DFT method has been calculated by using eqs 9–11. After obtaining the rate constant from forward reactions 1–3 and Gibbs free energy for the chemical reactions 1–3, the backward chemical rate for reactions 1–3 is calculated. For example, for chemical reaction 1, *k*_1_/*k*_–1_ = *K* = exp­(−Δ*G*_R1_/*RT*) and Δ*G*_R1_ is the defined Gibbs free energy.

DFT results for the Gibbs free energy and rate constants for reactions 1–3 on Ir_30_, Ir_20_Pt_10_, and Pt_30_ are presented in Table S1 of the Supporting Information.

Pt, Ir, and Ir–Pt nanoalloy structures with 30 atoms were geometrically optimized and modeled as catalysts supported on carbon nanotubes. The optimized structures for Pt (Figure S9A), Ir (Figure S9B), and Ir_80_Pt_20_ (Figure S9C) are provided in the Supporting Information.

The current density versus electrical overpotential for the hydrogen evolution reaction (HER) on the Pt–Ir nanoalloy is shown in Figure 12, where blue, orange, and magenta correspond to Ir_30_, Pt_30_, and Ir_20_Pt_10_, respectively. Current density was calculated based on eqs 4–7. The results indicate that even a small amount of Pt doping in the Ir nanoalloy significantly enhances the current density. A near-linear relationship between the current density and overpotential is observed, demonstrating the favorable electrocatalytic behavior of the nanoalloy.

**Figure 12:**
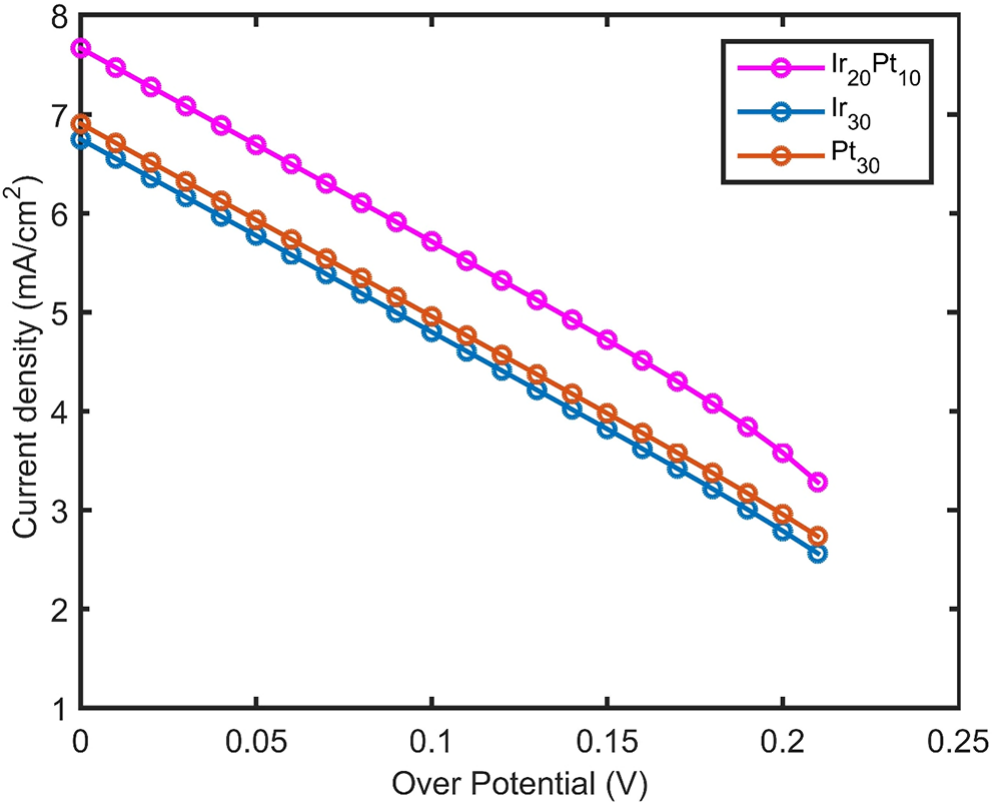
Current density versus electrical overpotential volt (V) for a total Ir atom number of 30 in the Ir nanocluster (Ir_30_), for a total Pt atom number of 30 in the Pt nanocluster (Pt_30_), and 20 Ir atoms and 10 Pt atoms in the Ir_20_ Pt_10_ nanoalloy for water electrolysis in alkaline media.

The lowest curve in Figure 12, corresponding to Ir nanoparticles alone, indicates the weakest catalytic activity for the HER in alkaline water electrolysis compared to the Pt NP and the Ir–Pt nanoalloy. Pure Pt exhibits slightly better activity than Ir, although it still falls short of optimal performance. The slope of the current density versus the overpotential curve shows that Pt NPs have a steeper slope than that of Ir, whereas the Ir–Pt nanoalloy demonstrates superior performance relative to both pure metals.

Density functional theory (DFT) calculations predict that at an overpotential of 50 mV in alkaline media, the current densities for the hydrogen evolution reaction (HER) are 5.5, 6.0, and 7.0 mA cm^–2^ for Ir, Pt, and Ir–Pt nanoalloys, respectively. The enhanced activity of the Ir–Pt alloy is likely due to two cooperative effects: electronic and geometric. The electronic density of states for the Ir–Pt nanoalloy increases when Ir is doped in Pt, and the quantum band gap in the Ir–Pt nanoalloy decreases with doping of Ir in the Pt nanoalloy as well. It gives better current density due to the better electron flow to the nonoccupied orbital for chemical species such as H in the HER, which happens on the surface of the Ir–Pt nanoalloy. Also, doping of Ir in the Pt nanoalloy facilitates migration of more atoms in the core to the surface. Then, geometric effects result from the alloyed structure, which generates additional active sites for the HER. Together, these electronic and geometric contributions significantly improve the current density and overall catalytic performance.

Our theoretical results for current density versus overpotential are consistent with the experimental findings reported for Ir–Pt nanoalloys in alkaline water electrolysis.^7^

According to the ref 7, the experimental results show that the electrical current density (*J*) for the hydrogen evolution reaction (HER) in alkaline media follows the order

J(Ir−Pt)>J(Pt)>J(Ir)

The trend and relative magnitude of the electrical current density obtained from our calculations are in agreement with these experimental results. However, in the present study, the nanoparticle sizes of both Pt and Ir are 30 atoms, which differ from the NP sizes reported in the experimental work. As a result, the calculated electrical current density of Pt is slightly higher than that of Ir with respect to the electrical overpotential, and the current densities of Pt and Ir are very close to each other.

## 5 Conclusions

Water electrolysis was systematically investigated using engineered electrodes based on Ir@SWCNT, Pt@SWCNT, and Ir–Pt nanoalloys supported on carbon-based porous substrates. The structural transition from crystalline (metallic) to amorphous phases in Ir nanoparticles (NPs) was examined in relation to the Tafel exchange current density during the hydrogen evolution reaction (HER) under acidic conditions. A nonmonotonic dependence of exchange current density on Ir particle size was observed, with a pronounced maximum at the crystalline-to-amorphous transition, highlighting the critical role of structural disorder in catalytic activity. The current density–overpotential characteristics were further evaluated for electrodes composed of Ir, Pt, and Ir–Pt nanoalloys in alkaline media. The Ir–Pt nanoalloy exhibits superior current density relative to monometallic Ir and Pt, indicating enhanced electrocatalytic performance toward the HER. In addition, hydrogen adsorption behavior on Ir@SWCNT, Pt@SWCNT, and Ir–Pt@SWCNT systems was analyzed. The results demonstrate that the Ir–Pt nanoalloy possesses a higher hydrogen adsorption capacity compared to pure metal nanoparticles. A temperature-dependent adsorption trend is also observed: Ir nanoparticles exhibit stronger hydrogen adsorption in the 300–320 K range, whereas Pt nanoparticles show enhanced adsorption in the 320–350 K range, reflecting differences in adsorption energetics and surface interactions. Extended molecular dynamics simulations were performed to investigate hydrogen diffusion and adsorption on Ir@SWCNT and Pt@SWCNT systems, based on DFT-parametrized interaction potentials for metal–hydrogen and metal–carbon interactions. The results indicate higher hydrogen diffusion and lower adsorption compared to simulations using the Lennard-Jones (LJ) potential. Furthermore, molecular dynamics simulations predict a lower number of adsorbed hydrogen molecules on Pt_*x*_Ir_1–*x*_@SWCNT compositions when using DFT-based potentials, in comparison with LJ interaction potentials. The trend of hydrogen adsorption as a function of the Pt content in Pt_*x*_Ir_1–*x*_@SWCNT differs between the two approaches: for *x* ≤ 0.5, the DFT-based results show an opposite trend compared to the LJ potential, whereas for *x* > 0.5, both methods exhibit a similar trend in hydrogen adsorption. The heat capacity associated with hydrogen adsorption on larger Ir@SWCNT nanoparticles exhibits nonmonotonic behavior with temperature, whereas for smaller nanoparticles, it increases monotonically. In general, the hydrogen diffusion coefficient on Ir@SWCNT is higher than that on Pt@SWCNT at smaller particle sizes. Density functional theory calculations were employed to analyze the vibrational density of states associated with the Tafel step of the hydrogen evolution reaction (HER) on Ir nanoparticles. As shown in Figure 10A,10B, chemical adsorption and the formation of Ir–H bonds lead to the splitting of an initially broad vibrational peak into two distinct peaks, indicating a strong interaction between hydrogen and the Ir surface.

## Supplementary Material



## Notes

Hydrogen adsorption on different sizes of Ir metal NPs on Ir@SWCNT, hydrogen adsorption on Ir@SWCNT (with an Ir size of 256 atoms) versus temperature, hydrogen adsorption on Pt@SWCNT (with a Pt size of 256 atoms) versus temperature, schematic presentation for simulation hydrogen adsorption on the Pt–Ir nanocatalyst with different Pt doping percentages, surface coverage of Ir@SWCNT for hydrogen adsorption versus temperature, free energy for reactions 1, 2, and 3 in the HER on different nanomaterials (Ir_30_, Ir_20_Pt_10_, Pt_30_), schematic presentation for Pt and Ir nanoparticles with sizes of 30 and Ir_20_Pt_10_, and fitting heat capacity versus temperature with polynomial series with order 4 for different sizes of Ir nanoparticles (PDF)

## References

[1] Krstajić N., Grgur B., Mladenović
† M. V., Mladenović N., Vojnović, Vojnović M., Jaks̆ić M. M. (1997). The determination
of kinetics parameters of the hydrogen evolution on TiNi alloys by *ac* impedance. Electrochemica Acta.

[2] Wang H., Min S., Wang Q., Li D., Casillas G., Ma C., Li Y., Liu Z., Li L.-J., Yuan J., Antonietti M., Wu T. (2017). Nitrogen-doped nanoporous carbon membranes with Co/CoP Janus-type
nanocrystals as hydrogen evolution electrode in both acidic and alkaline
environments. ACS Nano.

[3] Er D., Ye H., Frey N. C., Kumar H., Lou J., Shenoy V. B. (2018). Prediction
of Enhanced Catalytic Activity for Hydrogen Evolution Reaction in
Janus Transition Metal Dichalcogenides. Nano
Lett..

[4] Zheng Y., Jiao Y., Zhu Y., Hua Li L., Han Y., Chen Y., Du A., Jaroniec M., Zhang Qiao S. (2014). Hydrogen evolution
by a metal-free electrocatalyst, Hydrogen evolution by a metal-free
electrocatalyst. Nat. Commun..

[5] Zhu J., Hu L., Zhao P., Yoon Suk Lee L., Wong K-Yin. (2020). Recent Advances
in Electrocatalytic Hydrogen Evolution Using Nanoparticles. Chem. Rev..

[6] Nørskov J. K., Bligaard T., Logadottir A., Kitchin J. R., Chen J. G., Pandelov S., Stimming U. (2005). Trends in
the Exchange Current for
Hydrogen Evolution. J. Electrochem. Soc..

[7] Štrbac S., Smiljani M., Wakelin T., Potocnik J., Rakocevic Z. (2019). Hydrogen evolution
reaction on bimetallic Ir/Pt­(poly) electrodes in alkaline solution. Electrochim. Acta.

[8] El
Sawy E. N., Birss V. I. (2018). Nanoengineered Ircore@Ptshell Nanoparticles
with Controlled Pt Shell Coverages for Direct Methanol Electro-Oxidation. ACS Appl. Mater. Interfaces.

[9] Zhang S., Yin L., Wang S., Liu J.-C., Zhang Y., Wen Y., Zhang Q., Du Y. (2023). Ternary Rare Earth Alloy Pt3–xIrxSc
Nanoparticles Modulate Negatively Charged facilitate pH Universal
Hydrogen Evolution. ACS Nano.

[10] Anwar S., Khan F., Zhang Y., Djire A. (2021). Recent development
in electrocatalysts for hydrogen production through water electrolysis. Int. J. Hydrogen Energy.

[11] Gomez
Vidales A., Omanovic S. (2018). Evaluation of nickel-molybdenum-oxides
as cathodes for hydrogen evolution by water electrolysis in acidic,
alkaline, and neutral media. Electrochim. Acta.

[12] Wang S., Lu A., Zhong C.-J. (2021). Hydrogen
production from water electrolysis: role of
catalysts. Nano Convergence.

[13] Zeng K., Zhang D. (2010). Recent progress in alkaline water
electrolysis for hydrogen production
and applications. Prog. Energy Combust. Sci..

[14] Santos D. M. F., Sequeira C. A. C., Macciò D., Saccone A., Figueiredo J. L. (2013). Platinum–rare earth electrodes
for hydrogen evolution in alkaline water electrolysis. Int. J. Hydrogen Energy.

[15] Cardoso D. S. P., Amaral D. M. F., Santos B. S., Šljukić B., Sequeira C. A. C., Sequeira C., Macciò D., Macciò D., Saccone A. (2015). Enhancement of hydrogen evolution
in alkaline water electrolysis by using nickel-rare earth alloys. Int. J. Hydrogen Energy.

[16] Chen L., Dong X., Wang Y., Xia Y. (2016). Separating hydrogen
and oxygen evolution in alkaline water electrolysis using nickel hydroxide. Nat. Commun..

[17] Zheng Y., Jiao Y., Jaroniec M., Zhang Qiao Sh. (2015). Advancing
the Electrochemistry of the Hydrogen Evolution Reaction through Combining
Experiment and Theory. Angew. Chem., Int. Ed..

[18] Laursen A. B., Varela A. S., Dionigi F., Fanchiu H., Miller Ch., Trinhammer O. L., Rossmeisl J., Dahl S. (2012). Electrochemical Hydrogen
Evolution: Sabatier’s Principle and the Volcano Plot. J. Chem. Educ..

[19] Lao M., Li P., Jiang Y., Pan H., Xue Dou S., Sun W. (2022). From fundamentals
and theories to heterostructured electrocatalyst design: An in-depth
understanding of alkaline hydrogen evolution reaction. Nano Energy.

[20] Chauhan S. V., Joshi K. K., Pataniya P. M., Sumesh C. K. (2025). Advancing Industrial
Rate Current Density in Water Electrolysis for Green Hydrogen Production:
Catalyst Development, Benchmarking, and Best Practices. Sustainable Energy Fuels.

[21] Antolini E. (2014). Iridium As
Catalyst and Cocatalyst for Oxygen Evolution/Reduction in Acidic Polymer
Electrolyte Membrane Electrolyzers and Fuel Cells. ACS Catal..

[22] Taherkhani F. (2022). Hydrogen storage
via silver–aluminum bimetallic nanoparticle supported on different
shapes defect on carbon nanotube. Int. J. Hydrogen
Energy.

[23] Taherkhani F. (2024). Size and shapes
effect on structural and phonon density of state in Ir nanoparticle
and mechanical properties of Ir metal. J. Comp.
Mater. Sci..

[24] Torkashvand M., Gholivand M. B., Taherkhani F. (2015). Fabrication of an electrochemical
sensor based on computationally designed molecularly imprinted polymer
for the determination of mesalamine in real samples. Mater. Sci. Eng., C.

[25] Wang M., Zhou Y., Dong X., Xiao W., Liu Y., Qiao J. (2025). Dual skeleton network in situ-construction of alkaline
anion-exchange
membrane for boosting water electrolysis at low concentration of alkaline
solution. Process Saf. Environ. Prot..

[26] Shi J., Bao Y., Ye R., Zhong J., Zhou L., Zhao Z., Kang W., Aidarova S. B. (2025). Recent Progress
and Perspective of
Electrocatalysts for the Hydrogen Evolution Reaction. Catal. Sci. Technol..

[27] Li Z., Sun M., Zhang L., Zhou X. (2025). Recent Advances in the
Electrocatalytic Performance of Nanoporous Materials for Hydrogen
Evolution Reaction. Nanomaterials.

[28] González-Poggini S. (2024). Hydrogen Evolution
Descriptors: A Review for Electrocatalyst Development and Optimization. Int. J. Hydrogen Energy.

[29] Li J., Ma Y., Mu X., Wang X., Li Y., Ma H., Guo Z. (2025). Recent Advances
and Perspectives on Coupled Water Electrolysis for
Energy-Saving Hydrogen Production. Adv. Sci..

[30] Van
Der Heijden O., Heijden O., Park S., Park S.-H., Vos R. E., Eggebeen J. J. J., Koper M. T. (2024). Tafel Slope Plot
as a Tool to Analyze Electrocatalytic Reactions. ACS Energy Lett..

[31] Zhang L., Li W., Ren S., Zhang Y., Song W., Wang C., Lu X. (2025). Electronic Metal–Support
Interaction Induces Electron Deficiency
in Iridium for Promoted Ampere-Grade-Current-Density Electrocatalytic
Hydrogen Evolution. Chem. Sci..

[32] Tang H. (2024). Platinum Nanoparticles
Bonded with Carbon Nanotubes for High-Performance
Ampere-Level All-Water Splitting. ACS Omega.

[33] Shen T., Wang S., Zhao T., Hu Y., Wang D. (2022). Recent Advances
of Single-Atom-Alloy for Energy Electrocatalysis. Adv. Energy Mater..

[34] Conway B. E., Tilak, Basant V. (2002). Interfacial
Processes Involving Electrocatalytic Evolution and Oxidation of Hydrogen. Electrochim. Acta.

[35] Huang Z., Chen Z., Chen Z., Lv C., Humphrey M. G., Zhang C. (2014). Cobalt Phosphide Nanorods as an Efficient Electrocatalyst for the
Hydrogen Evolution Reaction. Nano Energy.

[36] Seh Z. W., Kibsgaard J., Dickens C. F., Chorkendorff I., Nørskov J. K., Jaramillo T. F. (2017). Combining Theory and Experiment in
Electrocatalysis: Insights into Materials Design. Science.

[37] Štrbac S., Smiljanić M., Wakelin T., Potočnik J., Rakočević Z. (2019). Hydrogen Evolution Reaction on Bimetallic
Ir/Pt­(poly) Electrodes in Alkaline Solution. Electrochim. Acta.

[38] McKone J. R., Sadtler B. F., Werlang C. A., Lewis N. S., Gray H. B. (2013). Ni–Mo
Nanopowders for Efficient Electrochemical Hydrogen Evolution. ACS Catal..

[39] Liu Q., Yang L., Sun P., Liu H., Zhao J., Ma X., Wang Y., Zhang Z. (2020). Ru Catalyst Supported on Nitrogen-Doped
Carbon Nanotubes as High-Efficiency Electrocatalysts for Hydrogen
Evolution in Alkaline Media. RSC Adv..

[40] Xu Y. (2009). The Hydrogen
Evolution Reaction on Single-Crystal Gold Electrodes. Int. J. Hydrogen Energy.

[41] Hussain S., Li H., Zhou M., Chen W. (2019). Fabrication of Robust
Hydrogen Evolution Reaction Electrocatalyst Using Ag_2_Se
by Vacuum Evaporation. Nanomaterials.

[42] Todorov I. T., Smith W., Trachenko K., Dove M. T. (2006). DL_POLY_3: new dimensions
in molecular dynamics simulations via massive parallelism. J. Mater. Chem..

[43] Bush I. J., Todorov I. T., Smith W. (2006). A DAFT DL_POLY distributed memory
adaptation of the Smoothed Particle Mesh Ewald method. Comput. Phys. Commun..

[44] Acharya C. K., Sullivan D. I., Turner C. H. (2008). Characterizing the
Interaction of
Pt and PtRu Clusters with Boron-Doped, Nitrogen-Doped, and Activated
Carbon: Density Functional Theory Calculations and Parameterization. J. Phys. Chem. C.

[45] Elvar Örn Jónsson Computational Approach to Electron Charge Transfer Reactions, PhD Thesis; Department of Physics, Technical University of Denmark 2013.

[46] Takamatsu A., Higashi M., Sato H. (2022). Free Energy and Solvation Structure
Analysis for Adsorption of Aromatic Molecules at Pt(111)/Water Interface
by 3D-RISM Theory. Chem. Lett..

[47] Akbarzadeh H., Yaghoubi H., Shamkhali A. N., Taherkhani F. (2014). CO Adsorption
on Ag Nanoclusters Supported on Carbon Nanotube: A Molecular Dynamics
Study. J. Phys. Chem. C.

[48] Taherkhani F., Akbarzadeh H., Abroshan H., Fortunelli A. (2012). Dependence
of Self-Diffusion Coefficient, Surface Energy, and Debye Temperature
on Size and Temperature for Aluminum Nanoclusters. Fluid Phase Equilib..

[49] Taherkhani F., Taherkhani F. (2022). Ir Nanocluster Shape Effects on Melting,
Surface Energy,
and Scaling Behavior of Self-Diffusion Coefficient near Melting Temperature. Comput. Mater. Sci..

[50] Metals – Melting Temperatures Reference Chart, Vize LLC | 13964 S. Wayside Houston, TX 77048 USA.

[51] Montero M. A., Fernández J. L., Gennero de Chialvo M.
R., Chialvo A. C. (2013). Kinetic
Study of the Hydrogen Oxidation Reaction on Nanostructured Iridium
Electrodes in Acid Solutions. J. Phys. Chem.
C.

[52] Mahmood J., Anjum M. A. R., Shin S. H., Ahmad I., Noh H. J., Kim S. J., Jeong H. Y., Lee J. S., Baek J. B. (2018). Encapsulating
Iridium Nanoparticles Inside a 3D Cage-Like Organic Network as an
Efficient and Durable Catalyst for the Hydrogen Evolution Reaction. Adv. Mater..

[53] Taherkhani F., Negreiros F. R., Parsafar G., Fortunelli A. (2010). Simulation
of vacancy diffusion in a silver nanocluster. Chem. Phys. Lett..

[54] Zheng Y., Jiao Y., Vasileff A., Qiao S.-Z. (2018). The Hydrogen
Evolution
Reaction in Alkaline Solution:From Theory, Single Crystal Models,
to Practical Electrocatalysts. Angew. Chem.,
Int. Ed..

