# Supplementary material for: Calculation Method for Hydrogen Evolution and Adsorption on Ir, Pt, and Ir–Pt Nanocatalysts Supported on Porous Carbon Electrodes in Water Electrolysis
Source: ACS Omega. 2026 May 8;11(19):29237–49. doi: 10.1021/acsomega.6c03700 (PMC13191503; doi:10.1021/acsomega.6c03700)
Supplement: Supplementary file 1 [file ao6c03700_si_001.pdf]

Calculational Method for Hydrogen Evolution and Adsorption on Ir, Pt, and Ir–Pt Nanocatalysts Supported on Porous Carbon Electrodes in Water electrolysis

Farid Taherkhani[1,2]*, Fabian Mauss

[1] *Departments of Thermodynamics and Thermal Process Engineering Brandenburg University of Technology, Cottbus, 03046, Germany*

[2] *Energiespeicher-und Energiewandlersysteme, Brandenburgische Technische Universität Cottbus–Senftenberg, Germany*

Corresponding Author: taherkha@b-tu.de, faridtaherkhani@gmail.com

Fig.S1

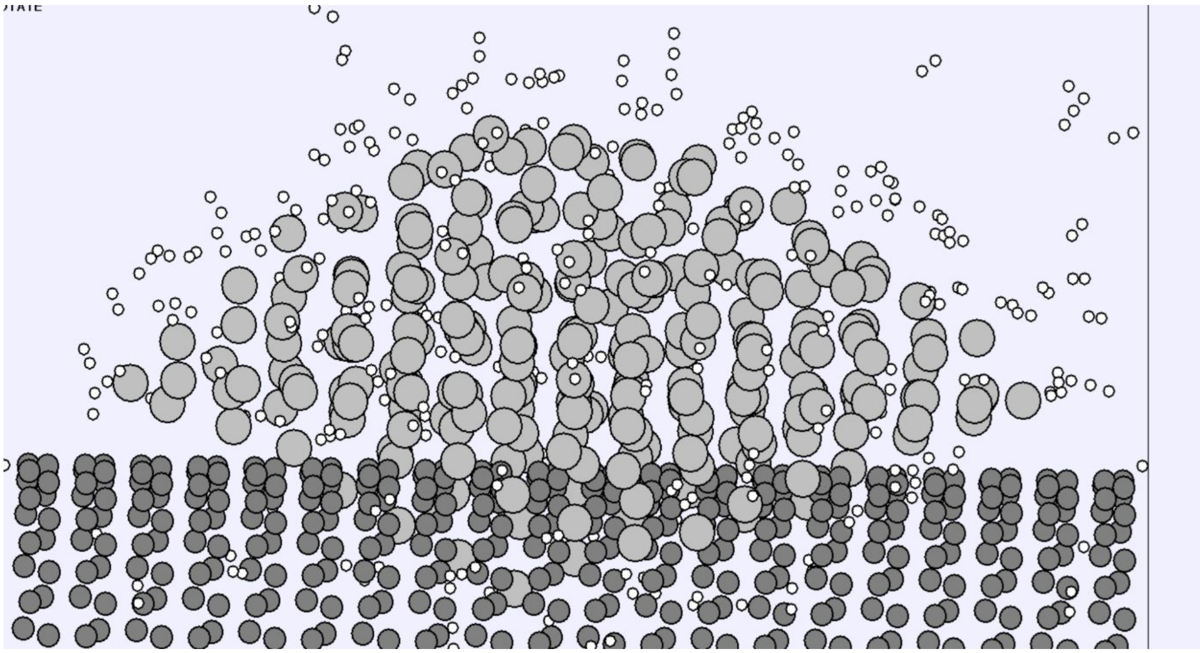

Fig.S1. Schematics presentation for Hydrogen adsorption on Ir metal nanoparticle with size N=256 which is supported on carbon nanotube

Fig.S.2

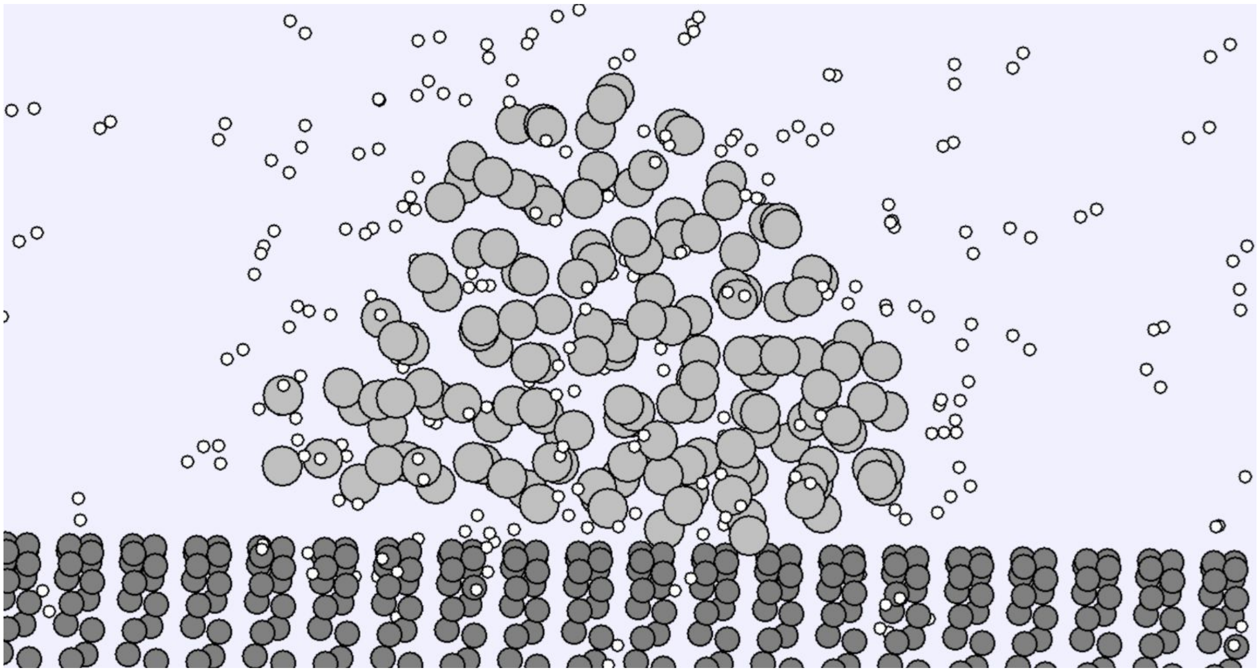

Fig.S2. Structure for hydrogen Adsorption on Ir nanoparticle with size N=156 which is supported on carbon nanotube

Fig.S3.

Fig.S4.

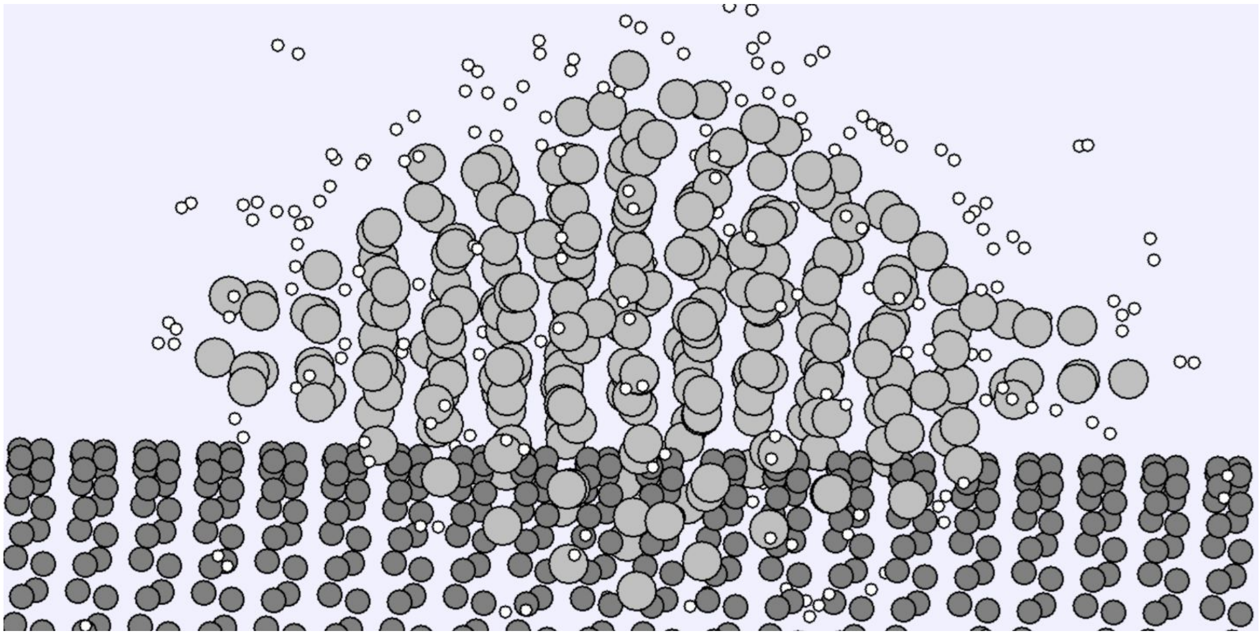

Fig.S3. Hydrogen Adsorption with initial hydrogen molecule number nH2=200 on Ir nanoparticle with size N=256 which is supported on carbon nanotube

Fig.S4.

Pressure Effect N=256, nH2=400

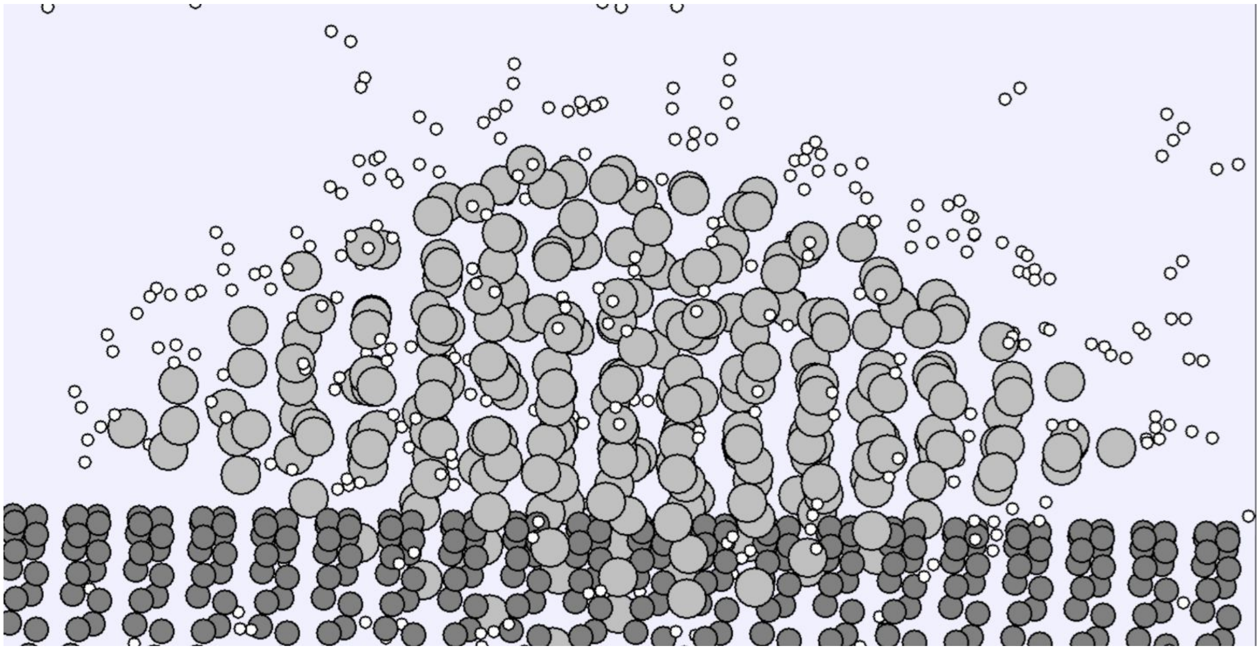

Fig.S4. Hydrogen Adsorption with initial hydrogen molecule number nH2=400 on Ir nanoparticle with size N=256 which is supported on carbon nanotube

Fig.S5.

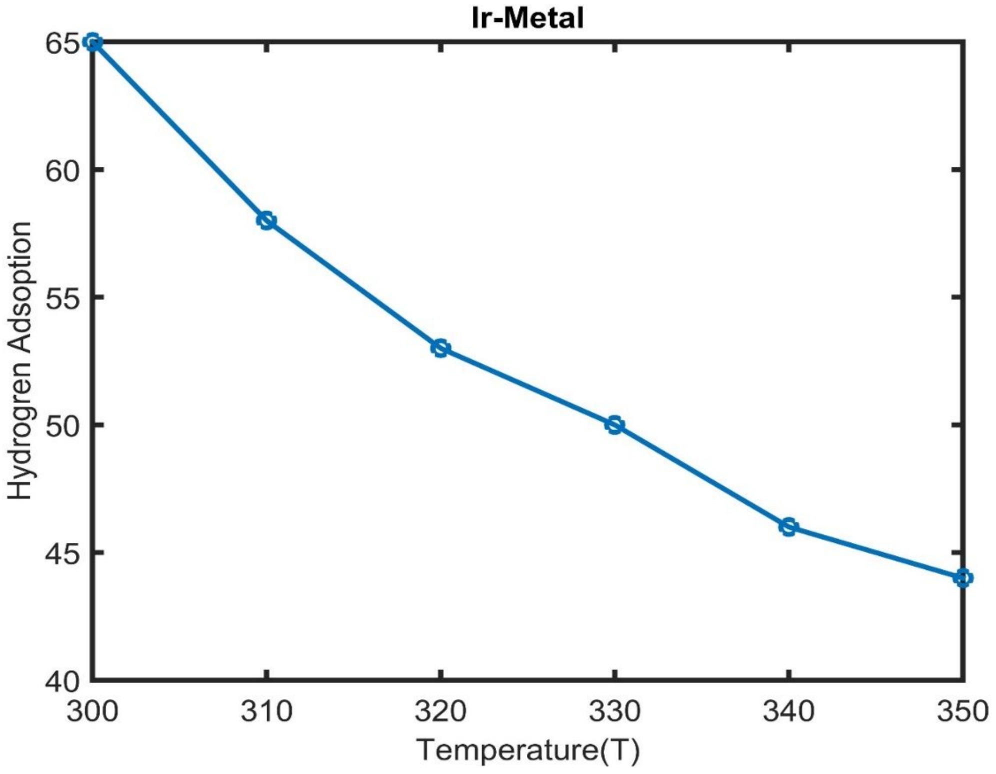

Fig.S5. Number of Hydrogen adsorption for Ir metal nanoparticle supported on carbon nanotube versus temperature for size 256.

Fig.S6.

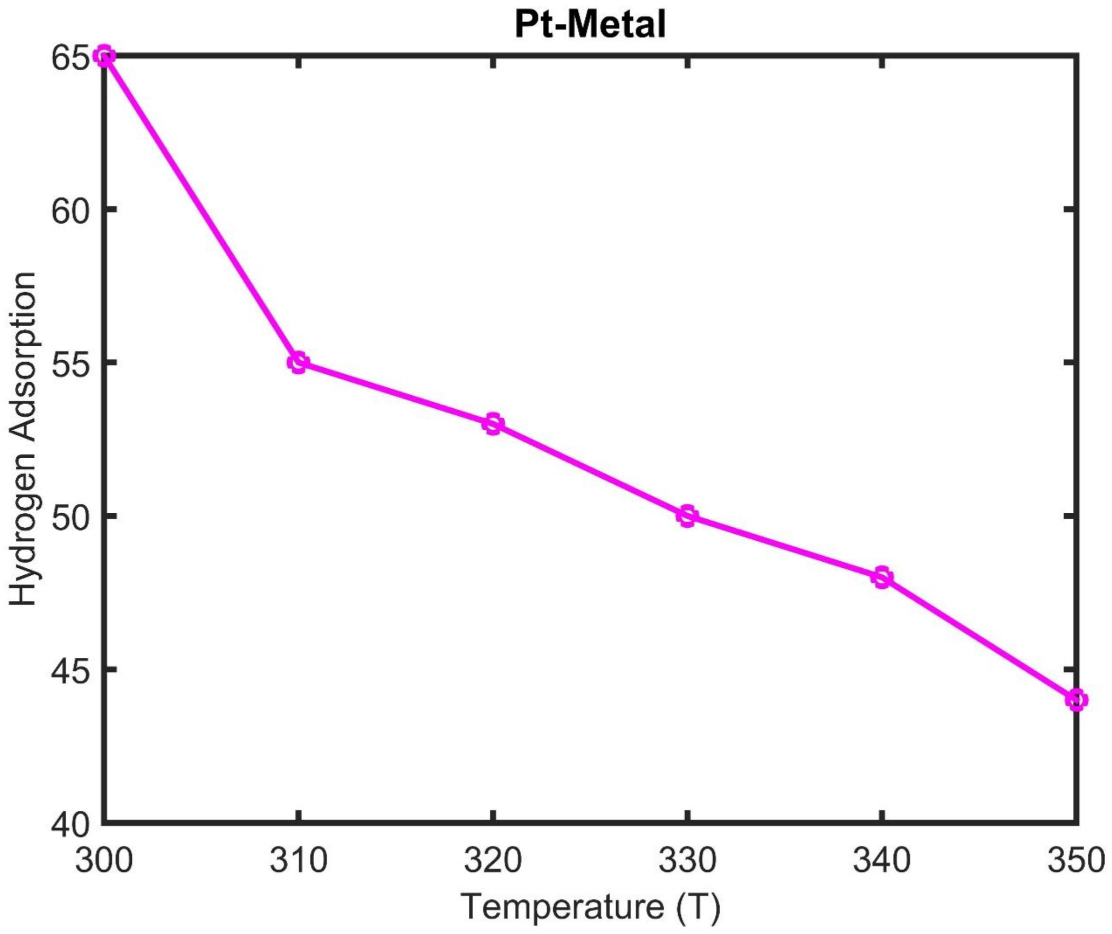

Fig.S6. Number of Hydrogen adsorption on Pt nanoparticle which is supported on carbon nanotube versus temperature

Fig.S7

20% Pt

50 % Pt

A

B

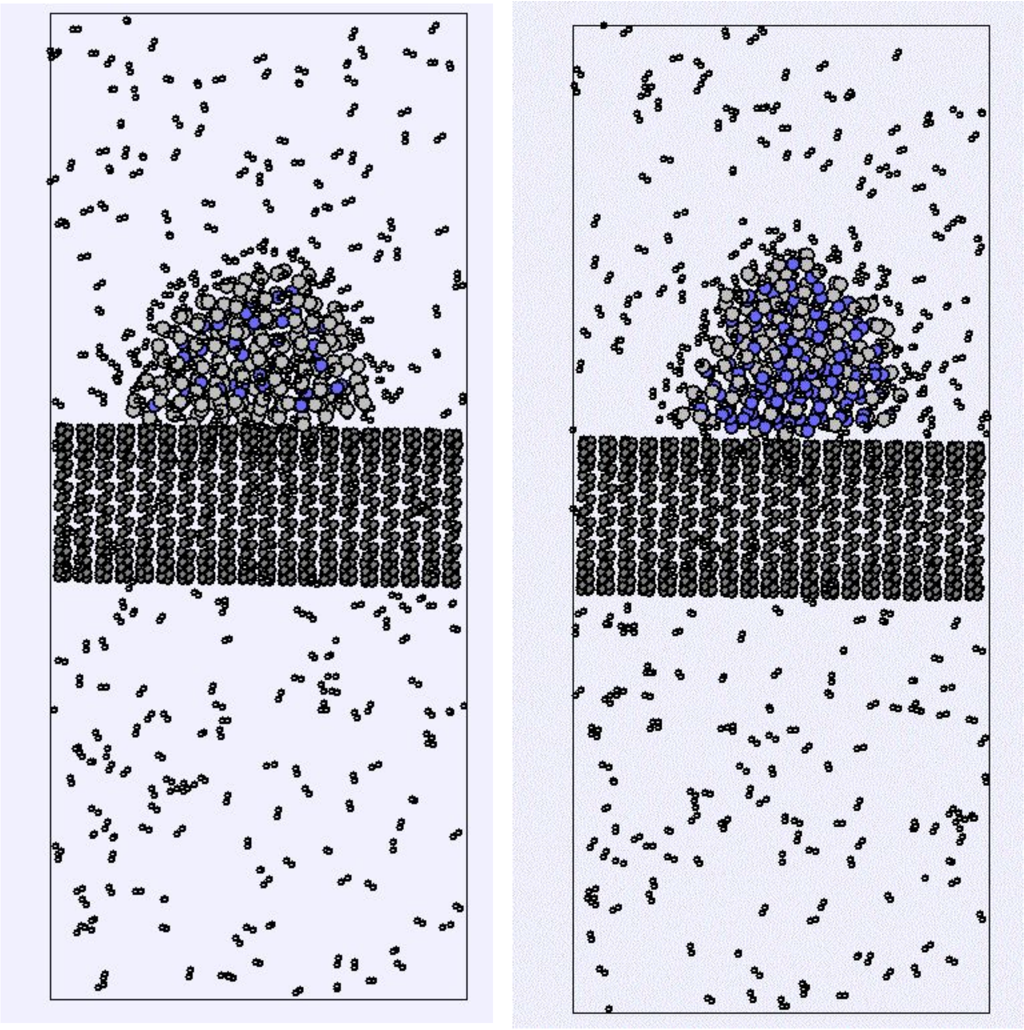

80 % Pt

100 % Pt

C

D

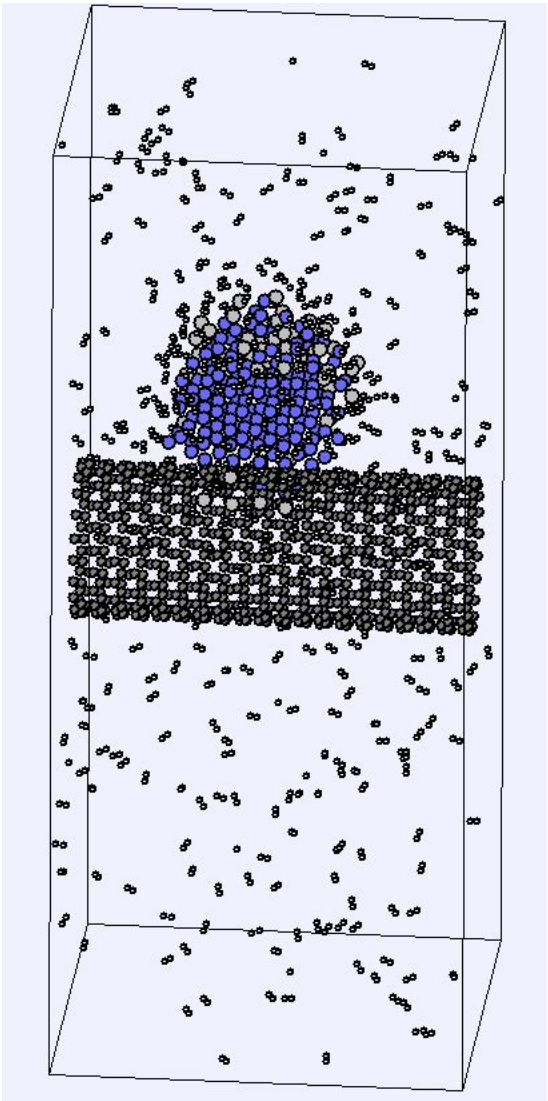

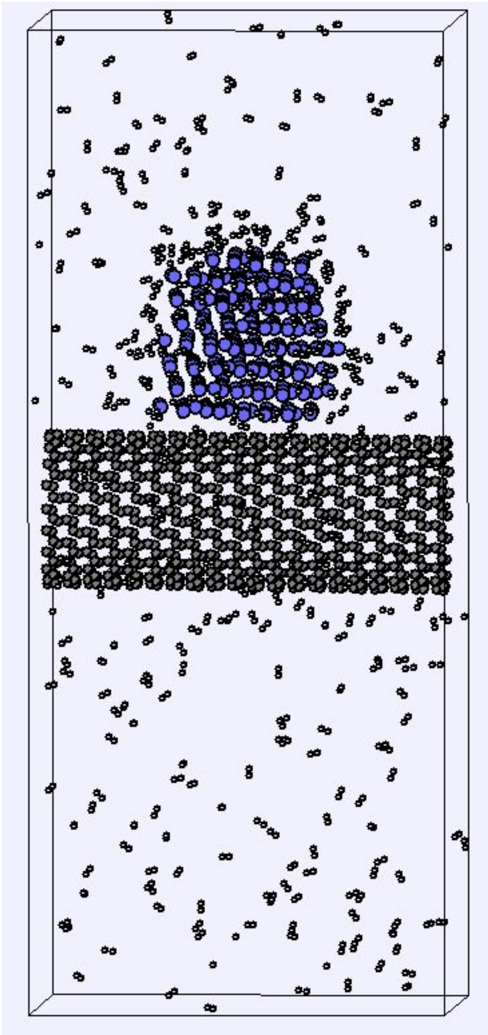

Fig.S7. A. Schematics presentation for simulation hydrogen adsorption on Pt-Ir nanocatalyst with Pt doping 20 percentage,

B. Schematics presentation for simulation hydrogen adsorption on Pt-Ir nanocatalyst with Pt doping 50 percentages.

C. Schematics presentation for simulation hydrogen adsorption on Pt-Ir nanocatalyst with Pt doping 80 percentages in Ir which is supported on carbon nanotube

D. Schematics presentation for simulation hydrogen adsorption on Pt nanoparticle which is supported on carbon nanotube

Fig.S8.

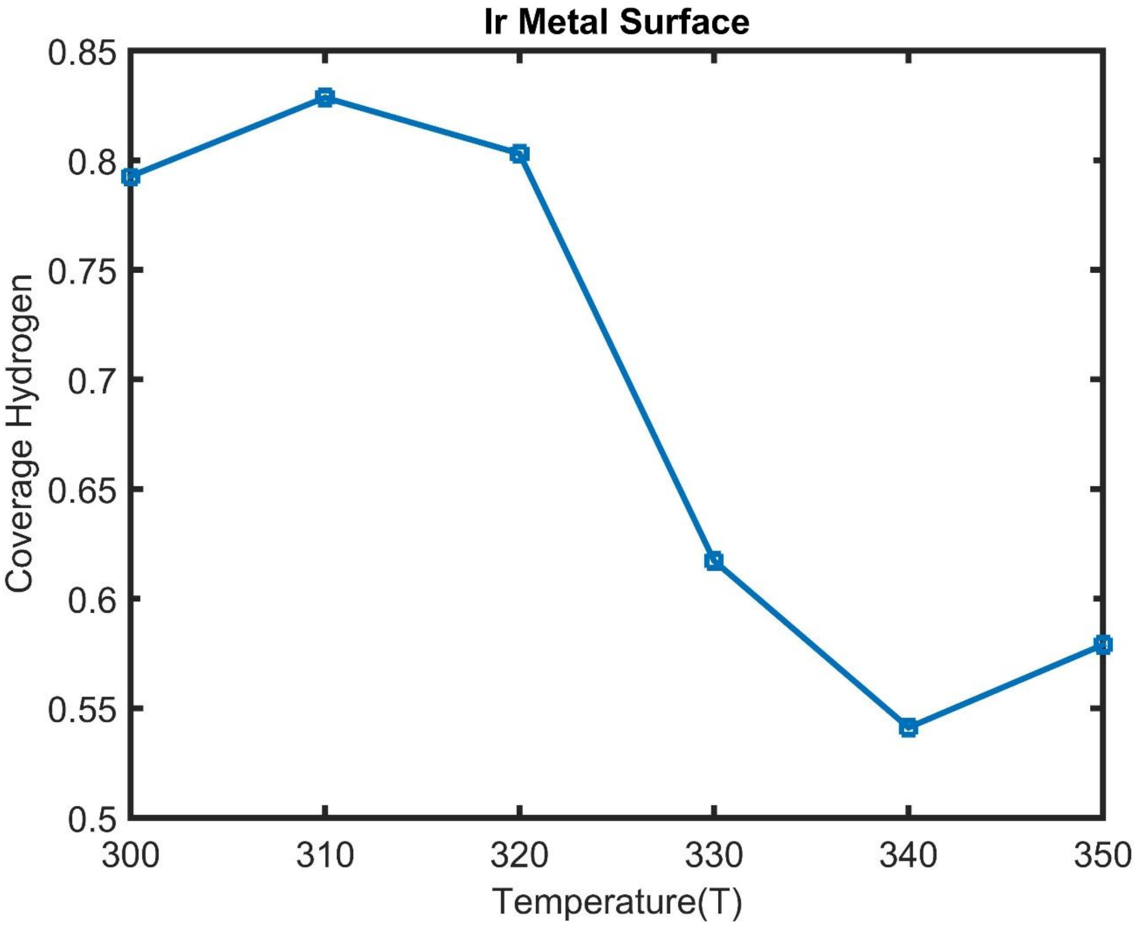

Fig.S8. Hydrogen coverage on Ir nanoparticle supported on carbon nanotube versus temperature

Table.1.S

Free Energy (J/mol)	$2.67*10^4$	$3.42*10^4$	$-1.56*10^4$
Reaction number (Ir_{30})	1	2	3

Free Energy (J/mol)	$1.62*10^5$	$-1.01*10^5$	$-1.51*10^5$
Reaction number ($Ir_{20}Pt_{10}$)	1	2	3

Free Energy (J/mol)	$4.09*10^4$	$2.0*10^4$	$-2.99*10^4$
Reaction number (Pt_{30})	1	2	3

For forward reaction 1-3 following rate constant is obtained.

(Ir_{30}) k1=9.8e-7; k2=2.5e-6; k3=4.3e-12;

($Ir_{20}Pt_{10}$) k1=9.95e-7; k2=2.1e-6; k3=4.1e-12;

(Pt_{30}) k1=9.85e-7; k2=2.6e-6; k3=4.7e-12;

Fig.S9

(A)

(B)

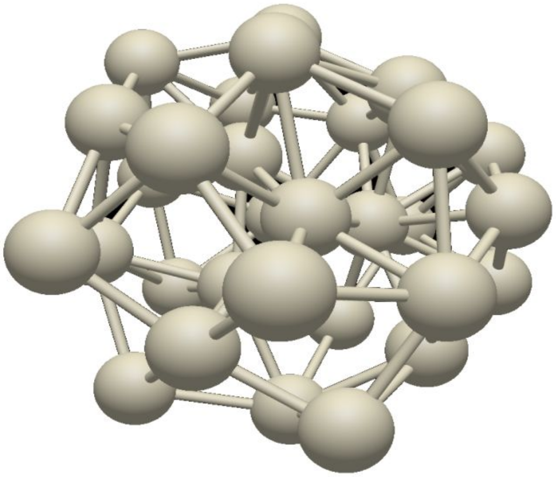 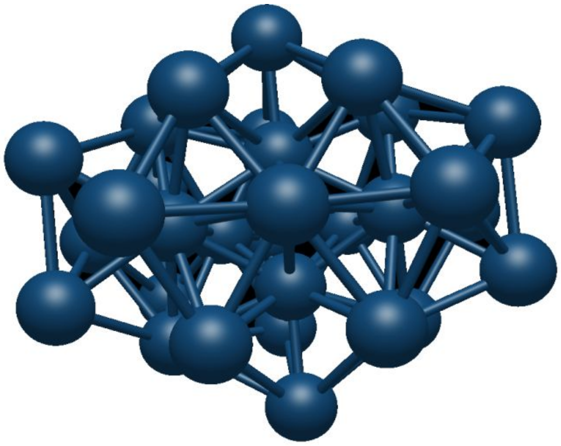

(C)

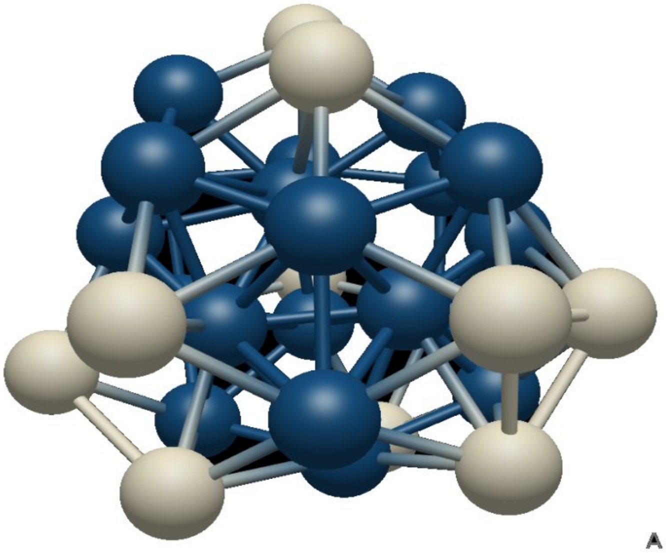

Fig.S9. A. Schematic presentation for Pt nanoparticle with Size 30

B. Schematic presentation for Ir nanoparticle with Size 30

C. Schematic presentation for Ir_{20}–Pt_{10} nanoparticle with total Size 30

Fitting Result.

Fitting heat capacity with polynomial series with order 4 for different size of Ir nanoparticle supported on carbon nanotube as $Cp(T)= C_1T^4+ C_2T^3+C_3T^2+C_4T+C_5$ in temperature range 300K ≤ T ≤ 400K

Table.2S.

N (Size of Ir nanoparticle)	C1	C2	C3	C4	C5	R-Square
50	$3.666*10^{-29}$	$-5.445*10^{-26}$	$2.993*10^{-23}$	$-7.266*10^{-21}$	$6.689*10^{-19}$	1
100	$-1.711*10^{-26}$	$2.40*10^{-23}$	$-1.263*10^{-20}$	$2.937*10^{-18}$	$-2.553*10^{-16}$	1
256	$3.053*10^{-27}$	$-4.049*10^{-24}$	$1.996*10^{-21}$	$-4.332*10^{-19}$	$3.49*10^{-17}$	1
